# Taohe Chengqi Decoction Attenuates Cognitive Impairment in Experimental Sepsis‐Associated Encephalopathy

**DOI:** 10.1002/cns.71106

**Published:** 2026-08-27

**Authors:** Xiaoru Yan, Jing Liu, Shu Chen, Yanan Zhu, Liang Yi, Xun Chen

**Affiliations:** ^1^ Department of Intensive Care Medicine Xiyuan Hospital of China Academy of Chinese Medical Sciences Beijing People's Republic of China; ^2^ Department of Medical Affairs Xiyuan Hospital of China Academy of Chinese Medical Sciences Beijing People's Republic of China; ^3^ Department of Laboratory Medicine Xiyuan Hospital of China Academy of Chinese Medical Sciences Beijing People's Republic of China; ^4^ Department of Pediatrics Xiyuan Hospital of China Academy of Chinese Medical Sciences Beijing People's Republic of China

**Keywords:** blood‐cerebrospinal fluid barrier, DUSP6, FOXO1, sepsis‐associated encephalopathy, Taohe Chengqi decoction

## Abstract

**Aims:**

Sepsis‐associated encephalopathy (SAE) is a clinical condition that is characterized by cognitive dysfunction induced by a systemic infection. This study was designed to assess the effect of the Taohe Chengqi decoction (THCQ) on SAE.

**Methods:**

THCQ (in doses ranging from low to high) was administered to rats before the CLP procedure. An evaluation of the survival, cognitive dysfunction, damage to cortical or hippocampal tissues, and intestinal permeability of septic rats was undertaken. Rat intestinal microvascular endothelial cells were exposed to lipopolysaccharide and cocultured with choroid plexus endothelial cells.

**Results:**

THCQ alleviated symptoms of SAE and reduced cerebral and hippocampal damage in rats afflicted with sepsis. THCQ reduced intestinal permeability and prevented blood‐cerebrospinal fluid barrier (BCSFB) disruption through systemic circulation. THCQ inhibited DUSP6 expression, and experimental overexpression of DUSP6 exacerbated intestinal barrier disruption and increased BCSFB permeability. The interaction between DUSP6 and FOXO1 mediated the dephosphorylation of FOXO1, increasing its nuclear translocation. Inhibition of FOXO1 reversed the effect of DUSP6 overexpression on SAE.

**Conclusion:**

By regulating the DUSP6/FOXO1 signaling, THCQ demonstrated protective effects on intestinal and BCSFB integrity, which were associated with improved cognitive outcomes in a rat model of SAE. These findings offer encouraging preclinical evidence with potential relevance.

## Graphical abstract

THCQ alleviates BCSFB permeability in sepsis‐associated encephalopathy by suppressing DUSP6, which blocks FOXO1 dephosphorylation and nuclear translocation. Therefore, THCQ protects against permeability changes induced by intestinal barrier disruption and highlights a potential therapeutic mechanism in SAE.

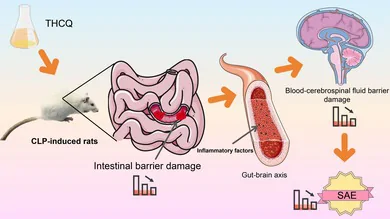

## 1 Introduction

Sepsis‐associated encephalopathy (SAE) is a severe neurological syndrome characterized by a diffuse brain dysfunction induced by sepsis, a life‐threatening condition caused by the body's dysregulated response to an infection [1]. Delirium often occurs at the onset of SAE, which is quickly followed by a rapid deterioration of mental status, characterized by memory and verbal deficits and possible onset of coma [2]. The potential mechanisms for the etiopathology of SAE include neuroinflammation, blood–brain barrier (BBB) disruption with endothelial/microglial activation, altered cerebral microcirculation, and alterations in neurotransmission [3]. Currently, there is no evidence‐based standard for diagnosing SAE, and symptomatic and supportive measures are the main direction for clinical management [4]. For instance, dexmedetomidine (DXM), an adrenergic α2‐receptor agonist, has been shown to prolong survival, reduce the density of apoptotic neurons, and mitigate spatial cognitive defects without altering motor activity in murine models of cecal ligation and puncture (CLP) [5]. Consequently, the modulation of endothelial cell dysfunction and the prevention of BBB permeability may emerge as viable therapeutic targets to enhance SAE outcomes.

In China, Traditional Chinese medicine has become a therapeutic option for the treatment of SAE because of its multitargeting effects and low toxicity and side effects [6]. Taohe Chengqi decoction (THCQ), a famous formula consisting of five Chinese medicines, including *Semen Persicae* (Peach Seed), *Radix et Rhizoma Rhei* (root and rhziome of Sorrel Rhubarb), *Ramulus Cinmomi* (Cassia Twig), *Radix Glycyrrhizae Preparata* (Prepared Liauorice Root), and *Natrii Sulfas1* (Sodium Sulfate), was first reported to alleviate renal fibrosis, possibly through inhibiting inflammatory response [7]. More recently, THCQ has been revealed to reduce mortality in septic mice, protect against CLP‐induced myocardial injury, and decrease systemic inflammatory response [8]. Inspired by these findings, we designed this study to dissect the role of THCQ in alleviating SAE‐induced cognitive deficit and the possible mechanism. Intriguingly, we identified that the inflammatory bowel disease pathway was enriched by target genes of the five herbs of THCQ. Ulcerative colitis led to gut vascular barrier impairment and fostered the recruitment of inflammatory cells to distal organs, thereby resulting in the alteration of choroid plexus (CP) permeability [9]. CP, a highly vascularized structure that contains endothelial, epithelial, immune, glial, and neuronal cells located in the ventricles of the brain, plays important roles in cerebrospinal fluid (CSF) production, aging, neuroimmune interactions, the pathogenesis of central nervous system (CNS) infections, and the constitution of a blood‐CSF barrier (BCSFB) [10]. However, whether THCQ regulates BCSFB through intestinal permeability in SAE remains unknown.

Our preliminary bioinformatics prediction and RNA‐sequencing (RNA‐Seq) revealed that dual specificity protein phosphatase 6 (DUSP6, also termed as mitogen‐activated protein kinase phosphatase 3) is a target of THCQ that is differentially expressed in the intestinal tissues of rats with CLP. The DUSP family phosphatases dephosphorylate both threonine/serine and tyrosine residues of their substrates [11]. Here, we assessed the protective activities of THCQ in the brains of the rats subjected to CLP and explored the role of DUSP6 in these activities.

## 2 Materials and Methods

More detail please find in the Methods S1.

## 3 Results

### 3.1 THCQ Alleviates the Cognitive Dysfunction Induced by SAE in Rats

THCQ is a classic formula comprising *Semen Persicae* (Peach Seed), *Radix et Rhizoma Rhei* (root and rhziome of Sorrel Rhubarb), *Ramulus Cinmomi* (Cassia Twig), *Radix Glycyrrhizae Preparata* (Prepared Liauorice Root), and *Natrii Sulfas1* (Sodium Sulfate) (Figure S1A). Firstly, we characterized THCQ by HPLC‐MS/MS; its positive and negative total ion current (TIC) diagrams are shown in Figure S1B, and its main components are shown in Table S1, 2 (Only the top 50 in terms of relative percentage of content are shown). Next, a rat model of sepsis was established by CLP surgery and pretreated with THCQ (low, moderate, and high doses) or the positive control, DXM (Figure S1C). THCQ at three doses did not have any effect on the survival of sham‐operated rats. By contrast, septic rats showed an increase in survival after treatment with moderate‐dose THCQ (M‐THCQ) and high‐dose THCQ (H‐THCQ) comparable to DXM treatment, but H‐THCQ did not further improve the efficacy (Figure S1D).

The learning and memory abilities of rats were evaluated using the Morris Water maze test. M‐THCQ treatment alone did not lead to significant changes in the learning and memory abilities of sham‐operated rats. Septic rats had an increase in the escape latency and the swimming distance during the exploration trial, whereas the escape latency and the swimming distance were shortened by the THCQ treatment at moderate and high doses, along with increased time spent in the platform quadrant in the exploration test (Figure S1E,F). In the open field test, septic rats spent less time in the center region of the open field, which was mitigated by THCQ pretreatment at moderate and high doses (Figure S1G). These findings indicate that THCQ was able to alleviate cognitive dysfunction in septic rats without toxic effects.

### 3.2 THCQ Alleviates the Disruption of the BBB and Reduces Neurological Damage in Septic Rats

**FIGURE 1:**
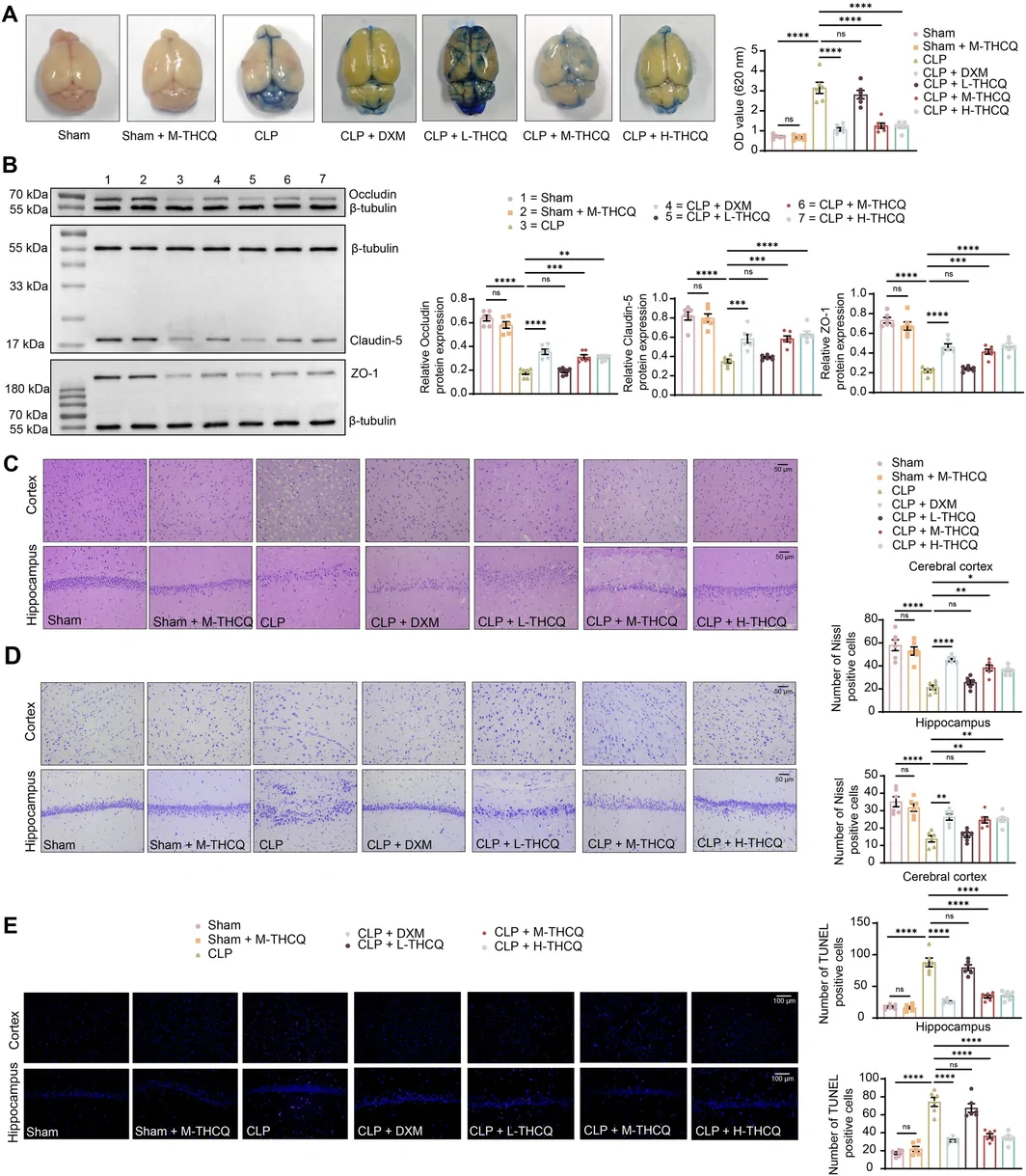
THCQ at moderate and higher doses alleviates BBB disruption and reduces neurological damage in septic rats. (A) Cerebral permeability in septic rats and THCQ‐treated rats was observed using Evans blue staining. (B) The protein expression of Occludin, Claudin‐5, and ZO‐1 in the brain tissues of rats was detected by western blot analysis. (C) Histopathological damage in the cortex and hippocampus of rats was observed using HE staining. (D) The number of Nissl‐positive neurons in the cortex and hippocampal tissues of rats was observed using Nissl staining. (E) Detection of apoptosis in rat cortex and hippocampal tissues by TUNEL (*n* = 6 per group). Data are shown as the mean ± SEM. The results were analyzed by one‐way ANOVA. *p < 0.05, **p < 0.01, ***p < 0.001, ****p < 0.0001.

Evans blue staining showed increased brain permeability in septic rats, which was alleviated by THCQ treatment at moderate and high doses and DXM (Figure 1A). Downregulation of Occludin, Claudin‐5, and ZO‐1 in brain tissues suggests disruption of the BBB in septic rats, whereas THCQ at moderate and high doses and DXM induced the expression of Occludin, Claudin‐5, and ZO‐1 (Figure 1B). HE staining showed that a large number of neurons in the cerebral cortex of septic rats were atrophied, with deeply stained nuclei and inflammatory cell infiltration. Hippocampal neurons were also damaged and solidified in CLP‐induced rats, with darker staining of nuclei, disorganized cellular arrangement, and inflammatory infiltration. M‐THCQ and H‐THCQ alleviated the pathological damage of the cerebral cortex and hippocampal tissues (Figure 1C). Neuronal nuclei in the cerebral cortex and hippocampus of rats in the sham group were dense and purplish‐blue, and the cytoplasm was enriched with Nissl bodies. By contrast, the cerebral cortex and hippocampus of the CLP‐induced rats showed augmented neuronal loss, disorganized and sparse arrangement of neurons, fuzzy or absent Nissl bodies, tissue disruption, and vacuolization of neurons. The M‐THCQ and H‐THCQ treatments enhanced the number of normal neurons and Nissl bodies, which in turn attenuated neuronal damage in the cerebral cortex and hippocampus (Figure 1D). Neuronal apoptosis was observed in the cerebral cortex and hippocampal tissues of septic rats, which was alleviated by the intervention of M‐THCQ and H‐THCQ (Figure 1E). Immunohistochemistry of the apoptosis‐related proteins in cerebral cortex and hippocampal tissues further confirmed neuronal apoptosis in septic rats, and M‐THCQ and H‐THCQ treatments reduced the expression of both (Figure S2A).

The cerebral cortex and hippocampal tissues of septic rats showed an increased infiltration of microglial cells (iBA1^+^) and astrocytes (GFAP^+^) (Figure S2B). ELISA showed increased release of IL‐6, TNF‐α, and IL‐1β (Figure S2C), and ICAM‐1 and VCAM‐1 in brain tissues of CLP‐induced rats (Figure S2D). M‐THCQ and H‐THCQ reduced the infiltration of microglia and astrocytes, and the levels of pro‐inflammatory cytokines in the brain tissue were greatly reduced. It was evident that THCQ did not change the brain tissue damage in the sham‐operated rats.

### 3.3 THCQ Inhibits the Disruption of the Intestinal Barrier by Reducing BCSFB

**FIGURE 2:**
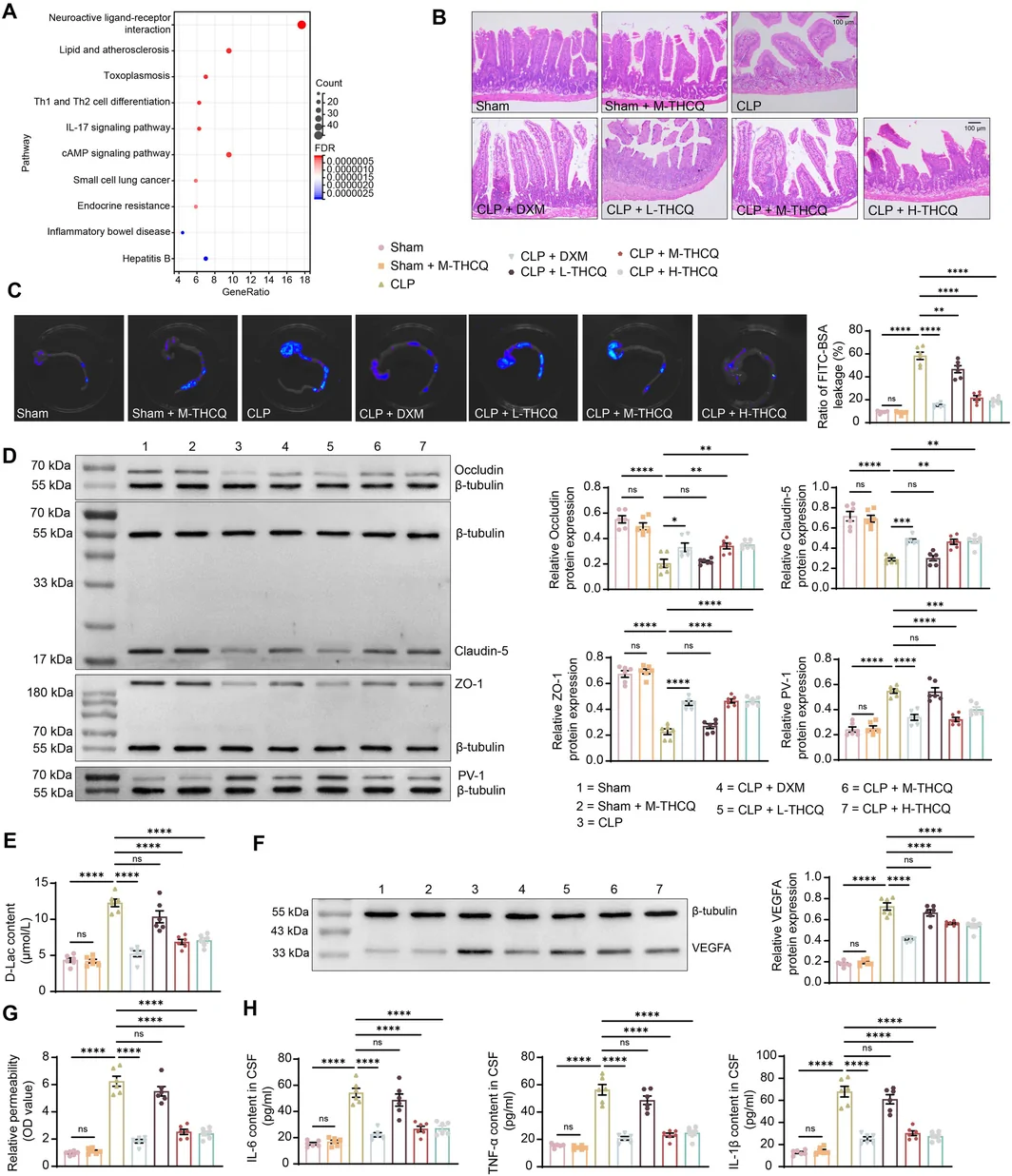
THCQ mitigates cerebral CP vascular barrier disruption by attenuating intestinal permeability. (A) KEGG pathway enrichment analysis of target proteins of the five ingredients of THCQ by the Hiplot tool. (B) Histopathological changes in the intestinal tract of septic or sham‐operated rats after treatment with THCQ by HE staining. (C) The intestinal permeability of rats was analyzed by FITC‐BSA permeation assay. (D) The protein expression of Occludin, Claudin‐5, ZO‐1, and PV‐1 in rat intestinal tissues was detected by western blot analysis. (E) Biochemical analysis of D‐lactic acid in the blood of rats. (F) The protein expression of VEGFA in rat intestinal tissues was detected by western blot analysis. (G) The permeation of dextran in the CSF of rats was examined using a 70‐kDa FITC‐dextran permeation assay. (H) Determination of IL‐6, TNF‐α, and IL‐1β in CSF of rats by ELISA (*n* = 6 per group). Data are shown as the mean ± SEM. The results were analyzed by one‐way ANOVA. *p < 0.05, **p < 0.01, ***p < 0.001, ****p < 0.0001.

To investigate the mechanism of THCQ alleviating SAE, we performed KEGG pathway enrichment analysis via the Hiplot database (https://hiplot.com.cn/home/index.html) after summarizing the gene targets of all five components in the HERB database (http://herb.ac.cn/). In the results of pathway enrichment analysis (Figure 2A), we found an interesting pathway: Inflammatory bowel disease. Therefore, is the effect of THCQ treatment on SAE attributable to the alleviation of intestinal permeability changes, which alleviates vascular barrier disruption in the cerebral CP and inhibits the spread of inflammatory responses?

As shown by HE staining, septic rats developed intestinal villous edema, inflammatory infiltration, and epithelial mucosal detachment, which were alleviated by M‐THCQ and H‐THCQ pretreatment (Figure 2B). The FITC‐BSA permeability of mesenteric microvessels was higher in the CLP group, while the treatment of M‐THCQ and H‐THCQ partially protected against the disruption of the intestinal barrier with a decrease in permeability (Figure 2C). The disruption of the intestinal barrier in septic rats and the protective effect of M‐THCQ and H‐THCQ on the intestinal barrier was also confirmed by detecting the expression of Occludin, Claudin‐5, ZO‐1, and PV‐1 in the intestinal tract (Figure 2D). Blood D‐lactate concentration was elevated in rats in the CLP group and decreased in M‐THCQ and H‐THCQ‐treated rats (Figure 2E). CLP also induced the expression of VEGFA in the intestines of rats, whereas M‐THCQ, H‐THCQ, and DXM reduced its expression (Figure 2F).

We then analyzed CP vascular barrier disruption in rats infused intravenously with 70 kDa FITC‐dextran and measured fluorescence infiltration into the CSF. Septic rats showed substantial FITC‐dextran infiltration into the CSF, whereas treatment with M‐THCQ and H‐THCQ decreased vascular barrier disruption in the CP (Figure 2G). Further measurements of inflammatory factors in the CSF also confirmed the infiltration of inflammatory factors in septic rats due to the disruption of the BCSFB, which was mitigated by M‐THCQ and H‐THCQ (Figure 2H). The integrity of BCSFB was disrupted in septic rats, which was partially mitigated by M‐THCQ and H‐THCQ (Figure S2E). VEGFA‐positive cells were increased in the CP of CLP‐induced rats, whereas the M‐THCQ and H‐THCQ treatment decreased VEGFA expression (Figure S2F). Finally, M‐THCQ and H‐THCQ partially restored CD31 expression in septic rats and alleviated barrier disruption (Figure S2G). Given the therapeutic effects of both moderate and high doses of THCQ in SAE rats, we chose the moderate dose of THCQ in the following study.

### 3.4 THCQ Inhibits Intestinal‐CP Vascular Barrier Permeability Impairment In Vitro

**FIGURE 3:**
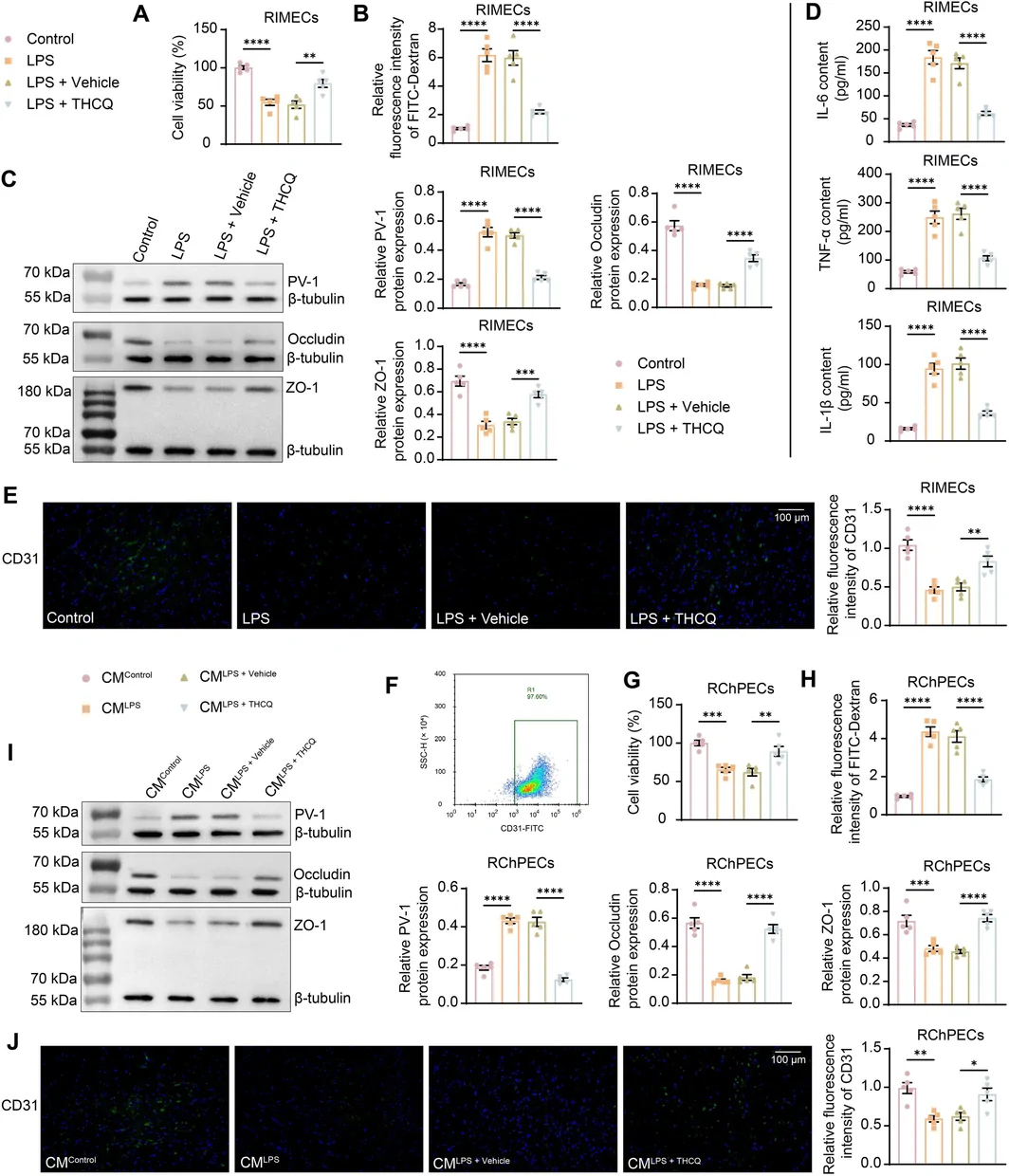
THCQ inhibits LPS‐induced inflammation and RIMEC and RChPEC permeability impairment in vitro. (A) The viability of RIMECs induced by LPS alone or simultaneously treated with THCQ was analyzed using CCK‐8 assays. (B) The permeability of RIMECs was analyzed by FITC‐dextran permeation assay. (C) The protein expression of Occludin, ZO‐1, and PV‐1 in RIMECs was detected by western blot analysis. (D) Determination of IL‐6, TNF‐α, and IL‐1β in RIMECs by ELISA. (E) Expression of CD31 in RIMECs was detected by immunofluorescence. (F) Positive expression of CD31 in extracted RChPECs was detected by flow cytometry. (G) The viability of RChPECs after culture with CM of RIMECs induced by LPS alone or simultaneously treated with THCQ. (H) The permeability of RChPECs after culture with CM of RIMECs was analyzed by FITC‐dextran permeation assay. (I) The protein expression of Occludin, ZO‐1, and PV‐1 in RChPECs after culture with CM of RIMECs was detected by western blot analysis. (J) Expression of CD31 in RChPECs after culture with CM of RIMECs was detected by immunofluorescence. (*n* = 5 per group). Data are shown as the mean ± SEM. The results were analyzed by one‐way ANOVA. *p < 0.05, **p < 0.01, ***p < 0.001, ****p < 0.0001.

Next, we treated RIMECs with LPS alone or simultaneously with THCQ. THCQ partially hampered the LPS‐induced reduction in cell viability (Figure 3A). LPS induced cell permeability, while THCQ protected the RIMECs from the LPS‐induced permeability disruption (Figure 3B). Meanwhile, THCQ treatment repressed the PV‐1 expression and IL‐6, TNF‐α, and IL‐1β release, while restoring Occludin and ZO‐1 protein expression (Figure 3C,D). As shown by immunofluorescence, THCQ treatment alleviated the CD31 repression by LPS exposure (Figure 3E).

We extracted RChPECs from neonatal rats and identified more than 95% of the cells expressing CD31 by flow cytometry (Figure 3F). CM from RIMECs was collected for RChPEC culture. After culturing with the CM, RChPECs exhibited suppressed viability and enhanced permeability, which were alleviated by CM from RIMECs treated with THCQ (Figure 3G,H). Assessment of the expression of the permeability marker PV‐1 with the tight junction markers in RChPECs also confirmed the mitigating effect of THCQ treatment in RIMECs on the disruption of permeability in RChPECs (Figure 3I). Finally, the decrease in CD31 expression in RChPECs cultured in CM from LPS‐treated RIMECs was reversed by THCQ in RIMECs (Figure 3J).

### 3.5 The Protective Effect of THCQ on the Intestinal Barrier Is Associated With DUSP6 Inhibition

We performed transcriptomic sequencing using intestinal tissues of rats induced by CLP (*n* = 3) and treated with THCQ (*n* = 3). A total of 1446 differentially expressed genes (Figure S3A) were visualized using the R software package “ggpubr” (v0.4.0). We then took the intersections of the differentially expressed genes with the gene targets of five components predicted by HERB, which showed only 42 intersections (Figure S3B). We found that the |LogFC value| of DUSP6 among the intersecting genes was the largest (LogFC = −5.43318358) (Figure S3C).

DUSP6 expression was upregulated in the intestines of septic rats, and THCQ inhibited the expression of DUSP6 (Figure S3D,E). Similarly, THCQ treatment reduced the expression of DUSP6 induced by LPS in RIMECs (Figure S3F,G).

### 3.6 Experimental DUSP6 Overexpression Impairs the Protective Effect of THCQ Against Intestinal Barrier Disruption and Exacerbates BCSFB Disruption

**FIGURE 4:**
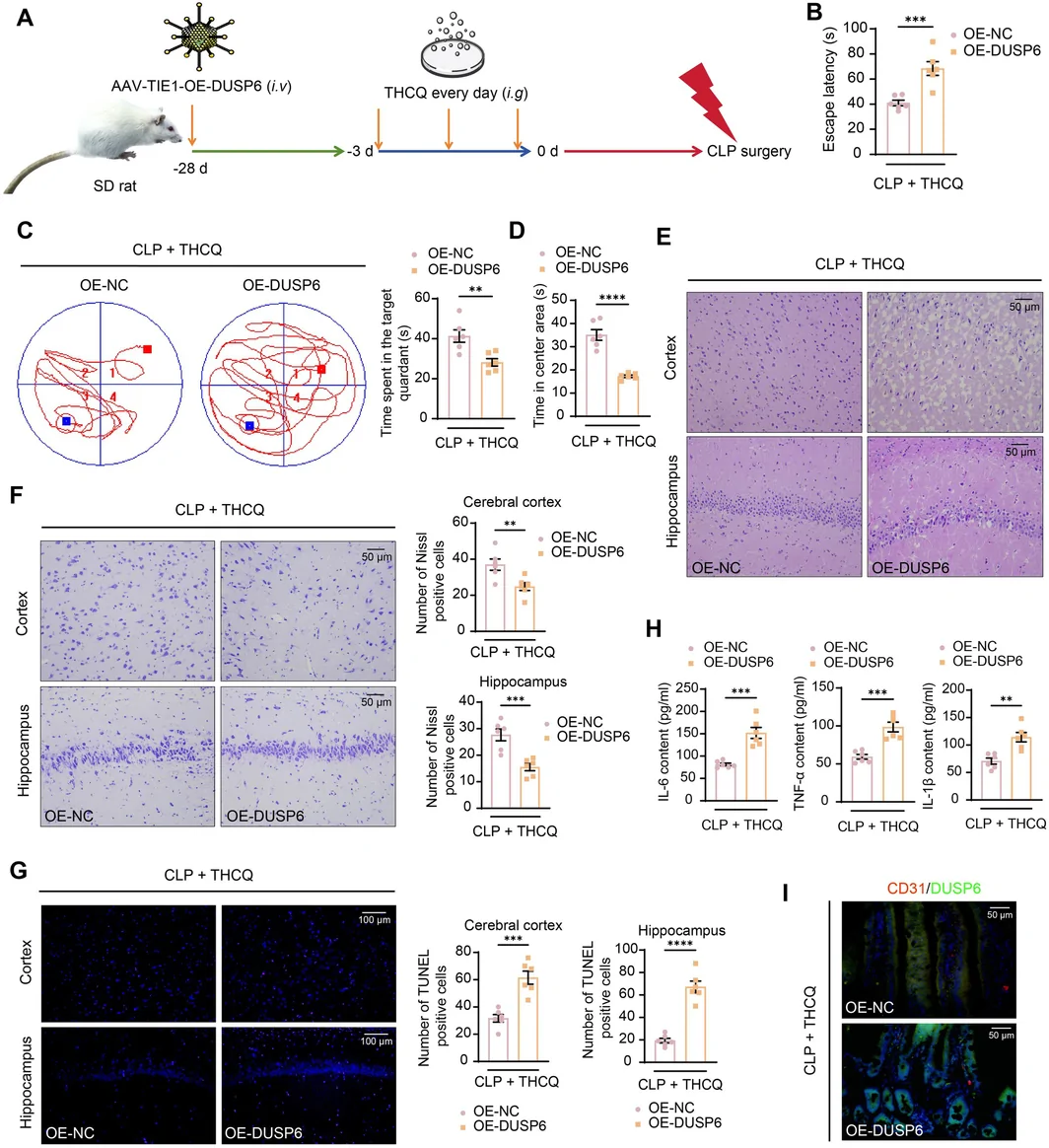
Specific overexpression of DUSP6 in endothelial cells exacerbates choroid plexus BBB disruption in SEA. (A) Timeline of the AAV administration, THCQ treatment, and CLP modeling in SD rats. (B) The escape latency of rats in the Morris water maze test. (C) Representative movement trajectories of rats and time spent in the platform quadrant in the exploration test in the Morris water maze test. (D) The residence time of rats in the center of the open field test. (E) Histopathological damage in the cortex and hippocampus of rats was observed using HE staining. (F) The number of Nissl‐positive neurons in the cortex and hippocampal tissues of rats was observed using Nissl staining. (G) Detection of apoptosis in rat cortex and hippocampal tissues by TUNEL. (H) Determination of IL‐6, TNF‐α, and IL‐1β in the brain tissues of rats by ELISA. (I) The expression and location of DUSP6 in microvascular endothelial cells of rat intestinal tissues were detected by dual‐labeling immunofluorescence. (*n* = 6 per group). Data are shown as the mean ± SEM. The results were analyzed by an unpaired *t*‐test. **p < 0.01, ***p < 0.001, ****p < 0.0001.

We performed experimental overexpression of DUSP6 in rats using AAV, followed by THCQ pretreatment and CLP (Figure 4A). Experimental DUSP6 overexpression increased cognitive dysfunction in rats, as reflected by a prolonged escape latency and an increase in swimming distance, along with reduced residence time in the platform quadrant (Figure 4B,C). In the open field test, rats had decreased time spent in the center of the open field (Figure 4D). Overexpression of DUSP6 increased damage in rat cortical and hippocampal tissues (Figure 4E), decreased the number of Nissl‐positive neurons (Figure 4F), and elevated cell apoptosis (Figure 4G), which was accompanied by an upregulation of the content of proinflammatory factors in the brain tissues of rats (Figure 4H).

Dual‐labeling immunofluorescence confirmed an increase in DUSP6 expression in rat intestinal endothelial cells (labeled by CD31) (Figure 4I). Experimental overexpression of DUSP6 increased intestinal permeability in rats (Figure S4A) and increased levels of D‐lactic acid in the blood of rats (Figure S4B). Interestingly, with the increase in intestinal permeability (Figure S4C), the BCSFB permeability was augmented (Figure S4D), and the levels of pro‐inflammatory cytokines in the CSF were elevated (Figure S4E). PV‐1 expression was increased in CP tissues, and ZO‐1 and CD31 expressions were significantly decreased (Figure S4F).

### 3.7 DUSP6 Repression by THCQ Blocks the Dephosphorylation and Nuclear Translocation of FOXO1

**FIGURE 5:**
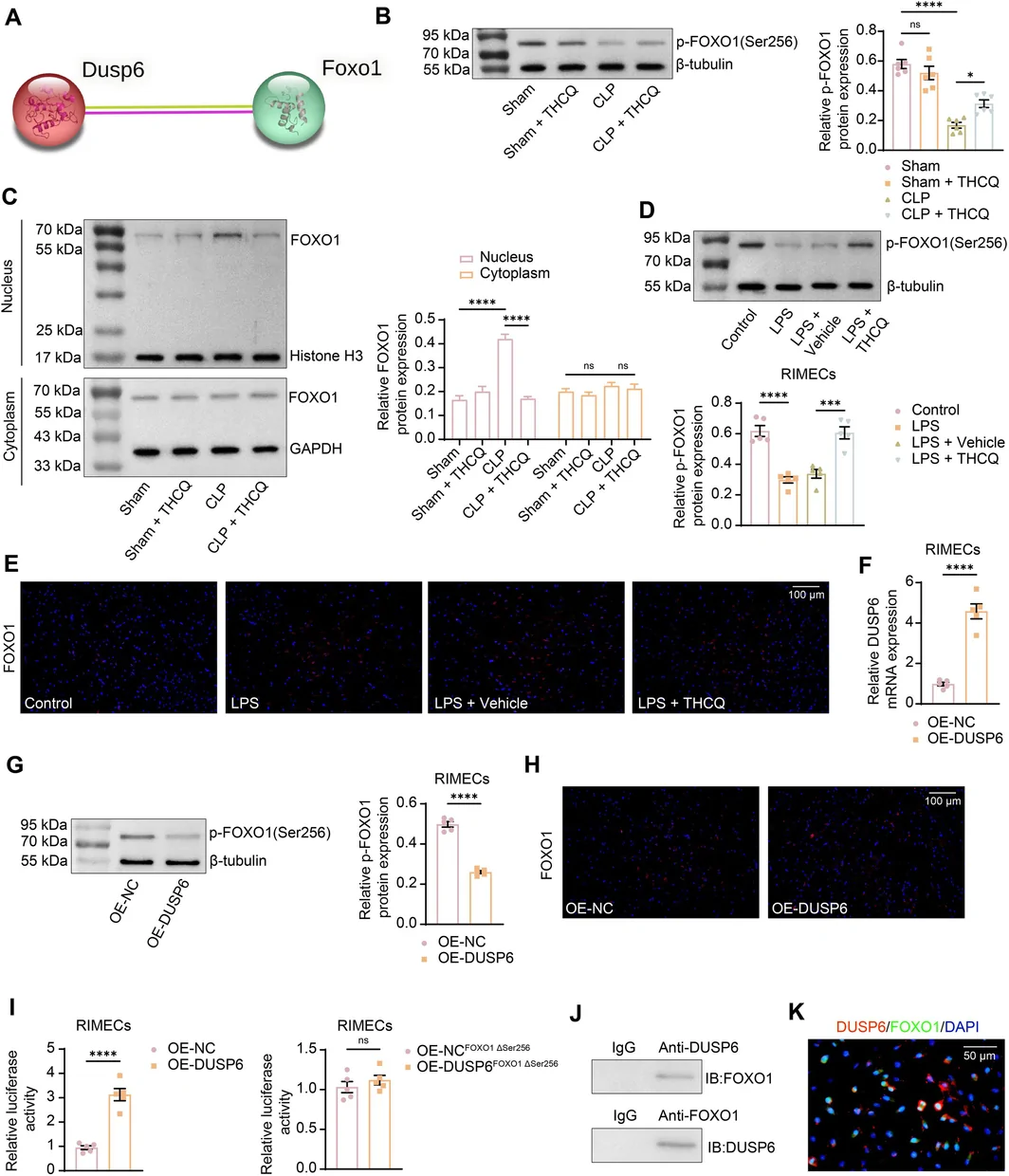
Inhibition of DUSP6 by THCQ blocks the dephosphorylation and nuclear translocation of FOXO1. (A) The protein interaction between DUSP6 and FOXO1 in rats was visualized using the String database. (B) Ser256 phosphorylation level of FOXO1 in intestinal tissues of septic rats and THCQ‐treated septic or sham‐operated rats was examined using western blot analysis (*n* = 6). (C) The nuclear and cytoplasmic expression of FOXO1 in intestinal tissues of septic rats and THCQ‐treated septic or sham‐operated rats was examined using subcellular fractionation assays (*n* = 6). (D) Ser256 phosphorylation level of FOXO1 in RIMECs induced with LPS alone or simultaneously treated with THCQ was examined using western blot analysis (*n* = 5). (E) Nuclear translocation of FOXO1 in RIMECs induced with LPS alone or simultaneously treated with THCQ was observed using immunofluorescence (*n* = 5). (F) DUSP6 mRNA expression after overexpression of DUSP6 in RIMECs was examined using RT‐qPCR (*n* = 5). (G) Ser256 phosphorylation level of FOXO1 in RIMECs overexpressing DUSP6 or not was examined using western blot analysis (*n* = 5). (H) Nuclear translocation of FOXO1 in RIMECs overexpressing DUSP6 or not was observed using immunofluorescence (*n* = 5). (I) FOXO1 transcriptional activity after overexpression of DUSP6 alone or with FOXO1 ΔSer256 deletion was examined using a dual‐luciferase reporter assay (*n* = 5). (J) The protein interaction between DUSP6 and FOXO1 in RIMECs was examined using a Co‐IP assay (*n* = 5). (K) Co‐localization of DUSP6 and FOXO1 by dual‐labeling immunofluorescence. Data are shown as the mean ± SEM. The results were analyzed using unpaired *t*‐tests (F, G, I), one‐way ANOVA (B, D), or two‐way ANOVA (C). *p < 0.05, ***p < 0.001, ****p < 0.0001.

A direct protein interaction of DUSP6 and FOXO1 in *Rattus norvegicus* was confirmed in the String database (https://string‐db.org/) (Figure 5A). The Ser256 phosphorylation of FOXO1 in the intestinal tissues of rats was examined. The phosphorylation level of FOXO1 in the intestinal tissues of septic rats was reduced, while the intervention of THCQ enhanced Ser256 phosphorylation of FOXO1 (Figure 5B). Increased nuclear expression of FOXO1 was found in the intestines of septic rats (Figure 5C). Subsequently, we also detected Ser256 phosphorylation and nuclear translocation of FOXO1 in RIMECs. LPS decreased phosphorylation of FOXO1 at Ser256 and induced nuclear translocation of FOXO1, and THCQ treatment led to the suppression of nuclear translocation of FOXO1 and an increase in the phosphorylation level of Ser256 (Figure 5D,E). RIMECs overexpressing DUSP6 were developed, and RT‐qPCR verified the successful overexpression of DUSP6 (Figure 5F). Experimental overexpression of DUSP6 decreased phosphorylation of FOXO1 at Ser256, along with an increase in nuclear translocation (Figure 5G,H). To clarify whether DUSP6 enhances the transcriptional activity of FOXO1 by regulating its nuclear translocation through dephosphorylation of Ser256, we used a luciferase reporter plasmid with a FOXO1‐binding site (pFOXO1‐TA‐Luc). Overexpression of DUSP6 induced luciferase activity of FOXO1, while when we performed deletion of Ser256 of FOXO1 (FOXO1 ΔSer256), DUSP6 did not affect the transcriptional activity of FOXO1 (Figure 5I). Co‐IP and dual‐labeling immunofluorescence assays showed that there was an interaction between DUSP6 and FOXO1 (Figure 5J,K).

### 3.8 Experimental Inhibition of FOXO1 Activity Alleviates the Symptoms of SAE in the Presence of DUSP6 Overexpression

**FIGURE 6:**
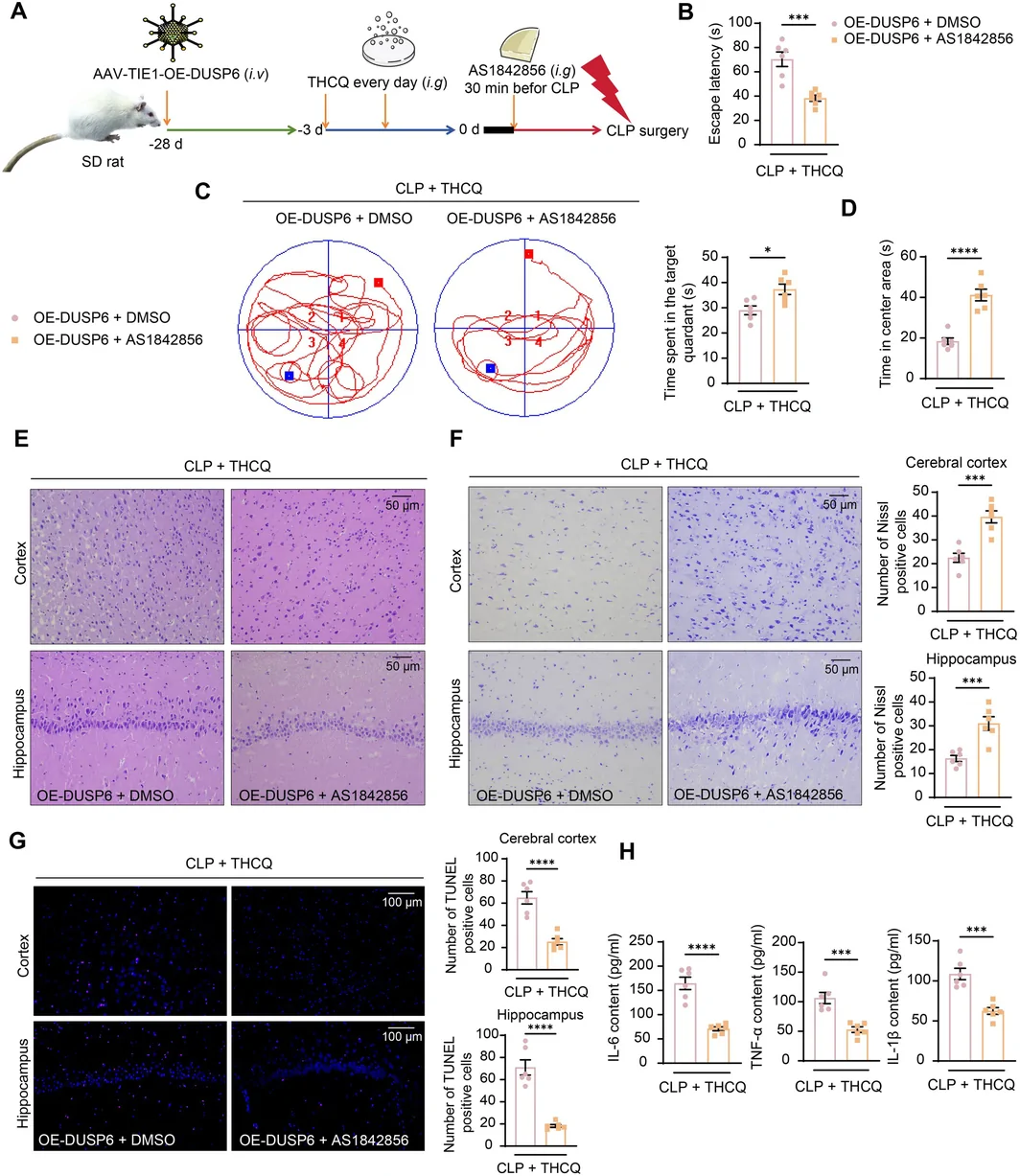
Inhibition of FOXO1 activity reverses choroid plexus‐cerebrospinal fluid barrier damage induced by overexpression of DUSP6 in SAE. (A) Timeline of the AAV administration, THCQ treatment, AS1842856 gavage, and CLP modeling in SD rats. (B) The escape latency of rats in the Morris water maze test. (C) Representative movement trajectories of rats and time spent in the platform quadrant in the exploration test in the Morris water maze test. (D) The residence time of rats in the center of the open field test. (E) Histopathological damage in the cortex and hippocampus of rats was observed using HE staining. (F) The number of Nissl‐positive neurons in the cortex and hippocampal tissues of rats was observed using Nissl staining. (G) Detection of apoptosis in rat cortex and hippocampal tissues by TUNEL. (H) Determination of IL‐6, TNF‐α, and IL‐1β in the brain tissues of rats by ELISA (*n* = 6 per group). Data are shown as the mean ± SEM. The results were analyzed by an unpaired *t*‐test. *p < 0.05, ***p < 0.001, ****p < 0.0001.

We inhibited FOXO1 activity by AS1842856 in rats overexpressing DUSP6, followed by THCQ treatment and CLP modeling (Figure 6A). Experimental inhibition of FOXO1 activity alleviated the exacerbation of cognitive dysfunction induced by DUSP6, with shorter escape latencies, shorter swimming distances, and longer residence time in the platform quadrant in Morris water maze tests (Figure 6B,C), and longer stay at the center in open field tests (Figure 6D). Experimental inhibition of FOXO1 activity alleviated the pathological damage in rat cortex and hippocampal tissues (Figure 6E), the number of Nissl bodies was elevated (Figure 6F), the neuronal apoptosis was downregulated (Figure 6G), and the levels of pro‐inflammatory cytokines in the brain tissues was significantly reduced (Figure 6H).

Experimental inhibition of FOXO1 activity decreased intestinal permeability (Figure S5A) and a decrease in blood D‐lactate levels (Figure S5B), protecting against disruption of the intestinal barrier induced by overexpression of DUSP6 (Figure S5C). At the same time, attenuated BCSFB impairment (Figure S5D) and a reduction of pro‐inflammatory cytokine content in the CSF were observed following inhibition of FOXO1 activity (Figure S5E). Our assessment of PV‐1, ZO‐1, and CD31 expression in the CP also confirmed that inhibition of FOXO1 activity mitigated disruption of the BCSFB (Figure S5F).

### 3.9 Experimental Inhibition of FOXO1 Activity Mitigates the Permeability of RIMECs and RChPECs

Similarly, we inhibited FOXO1 activity using AS1842856 on RIMECs after overexpression of DUSP6, followed by LPS and THCQ treatments. Overexpression of DUSP6 repressed RIMEC viability and enhanced permeability, which were reversed by AS1842856 (Figure S6A,B). Reduced expression of PV‐1 and elevated expression of Occludin and ZO‐1 in RIMECs also indicated that FOXO1 inhibition reversed the effect of overexpression of DUSP6 on the increase in cell permeability (Figure S6C). ELISA and immunofluorescence showed that overexpression of DUSP6 induced the content of IL‐6, TNF‐α, and IL‐1β, and repressed the CD31 staining in the RIMECs, while the inhibition of the activity of FOXO1 reversed the effect of DUSP6 (Figure S6D,E).

RChPECs were cultured with the CM of RIMECs after different treatments. The viability of RChPECs treated with CM of RIMECs expressing DUSP6 was significantly reduced, the cell permeability and the expression of PV‐1 were elevated, and the Occludin and ZO‐1 expression was reduced. By contrast, the inhibition of FOXO1 activity in RIMECs alleviated the damage and permeability of RChPECs (Figure S6F–H). Reduced CD31 expression in RChPECs induced by overexpression of DUSP6 in RIMECs was also ameliorated after FOXO1 activity was inhibited (Figure S6I).

We also knocked out DUSP6 in RIMECs using CRISPR. Western blot results showed that DUSP6 expression was absent after KO‐DUSP6 (Figure S7A). Further, we treated cells with LPS and THCQ, and KO‐DUSP6 further increased the therapeutic effect of THCQ (Figure S7B), decreased cell permeability (Figure S7C) and PV‐1 expression, and restored Occludin and ZO‐1 expression (Figure S7D). The pro‐inflammatory response of RIMECs induced by LPS was also decreased (Figure S7E), and CD31 expression was increased in cells with KO‐DUSP6 (Figure S7F). We then cultured RChPECs with the harvested CM, and knockout of DUSP6 attenuated RChPEC injury (Figure S7G) and permeability (Figure S7H), along with a downregulation of PV‐1 expression, and an increase in the expression of Occludin and ZO‐1 (Figure S7I). Finally, deletion of DUSP6 in RIMECs resulted in increased CD31 expression in RChPECs (Figure S7J).

## 4 Discussion

Our research demonstrated that THCQ alleviates neuroinflammation and cognitive dysfunction by modulating intestinal permeability‐induced BCSFB damage through the inhibition of DUSP6.

The important therapeutic effects of THCQ, whether by its classic five ingredients or added with more Chinese herbs, have been implicated in hepatic fibrosis [12], hepatic steatosis [13], diabetic cardiovascular complications [14], intracerebral hemorrhage‐induced brain injury [15], and sepsis‐induced lung injury [16] due to its anti‐inflammatory and immunomodulatory properties. The presence of SAE resulting from disrupted BBBs in patients is associated with notable cognitive impairment, including memory impairment and other indications of severe complications [17]. Our first in vivo finding was that THCQ pretreatment was beneficial in maintaining the integrity of the BBB after CLP surgery. Upregulation of ICAM‐1 during SAE, together with increased BBB permeability, might trigger circulating monocytes to adhere to the endothelium, and hypoxia‐ischemia caused by alterations in the brain microcirculation that occur in relatively early sepsis may be associated with SAE through the upregulation of inflammatory cytokines [18]. Consistently, we observed that the contents of these pro‐inflammatory cytokines and the infiltration of inflammatory cells were repressed in response to THCQ pretreatment as well.

Giridharan et al. demonstrated that the 24‐h and 10‐day CLP were capable of modulating several parameters of the gastrointestinal microbiota, including the length of the villi and crypts in the gut, the alpha and beta diversities of the microbial communities, and the levels of fecal short‐chain fatty acids [19], which was in line with our prediction results that the targets of five ingredients of THCQ were enriched to the inflammatory bowel disease pathway in addition to the classic inflammatory pathways. During sepsis, activation of immune cells increases cytokine production, resulting in increased lactate production, and tissue hypoxia induced by microvascular injury further exacerbates lactate production [20]. The disruption of the intestinal barrier was partially alleviated in rats pre‐administered with THCQ, and the D‐lactic acid contents and the expression of VEGFA, along with the endothelial permeability marker PV‐1, were all repressed. The BCSFB, an understudied brain barrier site compared to the BBB, is a potential therapeutic target to improve the delivery of CNS therapeutics and provide brain protection measures [21]. The CP is the location of a BCSFB that separates the CSF from the bloodstream in the CP endothelium [22, 23]. Considering the important findings by Carloni et al. that a vascular barrier in the CP responded to intestinal inflammation through bacteria‐derived LPS [9], we wonder whether the easing effects of THCQ on BBB and cognitive dysfunction might be related to BCSFB maintenance related to an alleviated intestinal barrier. The novel in vitro culture system comprising RChPECs and CM of RIMECs after different treatments verified this hypothesis.

As for the molecular mechanism, we identified DUSP6 as a key gene regulated by THCQ and differentially expressed in the intestinal tissues of THCQ‐treated rats. Interestingly, DUSP6 deficiency has been reported to strengthen the junctions and extend the microvilli, thereby enhancing colonic barrier integrity in DSS‐induced colitis [24]. Adolescent intermittent alcohol consumption has been demonstrated to increase DUSP6 expression, and the infusion of AAV‐DUSP6‐shRNA has been shown to restore dendritic spine density and postsynaptic density thickness, thereby repairing adult cognitive impairment caused by chronic alcohol exposure [25]. Here, by experimentally overexpressing DUSP6 using AAV vectors with an endothelial cell‐specific TIE1 promoter, we observed that the THCQ effects were largely compromised in the brain tissues and intestinal tissues. DUSP6‐mediated dephosphorylation of FOXO1 at Ser256 has been demonstrated to promote its nuclear translocation and recruitment to the promoters of gluconeogenic genes [26]. FOXO1 activation is known to include its dephosphorylation and nuclear translocation [27]. Our in vitro studies using RIMECs also validated the dephosphorylation and nuclear translocation of FOXO1 by DUSP6. FOXO1 expression was elevated in oxygen/glucose deprivation‐induced brain MECs, and its knockdown enhanced cell viability and repressed permeability of brain MECs [28]. Selective inhibition of FOXO1 activation, AS1842856, alleviated cardiac dysfunction, pathological damage, mitophagy, and mitochondrial dynamics in diabetic db/db mice [29]. Consistently, AS1842856 reversed the impact of DUSP6 overexpression and maintained the protective effects of THCQ.

While the results suggest a potential association between peripheral barrier integrity and central nervous system function, further studies employing targeted interventions (e.g., microbiota modulation) are necessary to substantiate the causal sequence underlying these observations. Furthermore, the present study was conducted using a rat CLP model of SAE. The pathophysiology of SAE in patients is more complex, and differences in species, experimental conditions, and clinical heterogeneity may limit translational relevance. Subsequent studies employing diverse animal models and, ultimately, clinical cohorts will be necessary to ascertain whether the protective effects observed here extend to human SAE.

In summary, the present study indicates that the administration of THCQ resulted in a demonstrable alleviation of cognitive dysfunction in a rat model of SAE. The protective effects observed were associated with modulation of DUSP6/FOXO1 signaling, resulting in enhancements in intestinal barrier and BCSFB integrity.

## Author Contributions

X.Y. and J.L. performed in vitro and in vivo experiments, and data analysis; S.C. performed in vivo experiments; Y.Z. performed in vitro experiments and contributed to the study concept and research design; L.Y. and X.C. wrote and reviewed the manuscript. All authors contributed to the article and approved the submitted version.

## Funding

This work was supported by the National Natural Science Foundation of China (No. 82204913); the China Academy of Chinese Medical Sciences outstanding young science and technology talent training program (No. ZZ16‐YQ‐012); and the Haidian District traditional Chinese medicine service system construction project (No. 010990015).

## Ethics Statement

The study protocol was approved by the Medical Ethics Committee of Xiyuan Hospital of China Academy of Chinese Medical Sciences (approval number: 2025XLC095) and complies with the ARRIVE guidelines.

## Conflicts of Interest

The authors declare no conflicts of interest.

## Supporting information


**Figure S1:** THCQ alleviates cognitive dysfunction in rats with SAE. (A) Ingredients of THCQ. (B) Positive and negative TIC plots of THCQ identified by HPLC‐MS/MS. (C) Timeline of the THCQ treatment and CLP modeling in SD rats. (D) The 96‐h survival of rats pretreated with THCQ and undergoing sham or CLP surgery was analyzed using the log‐rank test. (E) The escape latency of rats in the Morris water maze test. (F) Representative movement trajectories of rats and time spent in the platform quadrant in the exploration test in the Morris water maze test. (G) The residence time of rats in the center of the open field test. (*n* = 6 per group). Data are shown as the mean ± SEM. The results were analyzed by one‐way ANOVA. *p < 0.05, **p < 0.01, ***p < 0.001, ****p < 0.0001.
**Figure S2:** THCQ attenuates neuronal apoptosis, reduces inflammatory response, and mitigates gut–brain axis‐induced CP vascular barrier disruption in septic rats. (A) Expression of Bax and Caspase‐3 in the cortex and hippocampus of rats was detected by immunohistochemistry. (B) Expression of iBA1 and GFAP in cortical and hippocampal tissues of rats was detected by immunohistochemistry. (C) The levels of pro‐inflammatory cytokines IL‐1β, IL‐6, and TNF‐α in the brain tissues of rats were assessed using ELISA. (D) The levels of ICAM‐1 and VCAM‐1 in the brain tissues of rats were assessed using ELISA. (E) Expression of PV‐1, Claudin‐5, and ZO‐1 in the choroid plexus of rats was detected by immunofluorescence. (F) Expression of VEGFA in the choroid plexus of rats was detected by immunofluorescence. (G) Expression of CD31 in the choroid plexus of rats was detected by immunofluorescence. (*n* = 6 per group). Data are shown as the mean ± SEM. The results were analyzed by one‐way ANOVA. **p < 0.01, ***p < 0.001, ****p < 0.0001.
**Figure S3:** Protective effect of THCQ on the intestinal barrier is associated with inhibition of DUSP6. (A) The volcano maps of differentially expressed genes screened out from RNS‐seq analysis using intestinal tissues of CLP‐induced and THCQ‐treated rats (*n* = 3). (B) The intersection of targets of THCQ ingredients predicted by the HERB database and the differentially expressed genes in intestinal tissues. (C) The heatmap of 42 intersecting genes. (D) Expression and location of DUSP6 in microvascular endothelial cells of rat intestinal tissues were detected by dual‐labeling immunofluorescence (*n* = 6). (E) The mRNA expression of DUSP6 in the intestinal tissues of rats was examined using RT‐qPCR (*n* = 6). (F) The protein expression of DUSP6 in RIMECs was detected by Western blot analysis (*n* = 5). (G) The mRNA expression of DUSP6 in RIMECs was detected by RT‐qPCR (*n* = 5). Data are shown as the mean ± SEM. The results were analyzed by one‐way ANOVA. ***p < 0.001, ****p < 0.0001.
**Figure S4:** Specific overexpression of DUSP6 in endothelial cells impairs the protective effect of THCQ against intestinal barrier disruption. (A) The intestinal permeability of rats was analyzed by FITC‐BSA permeation assay. (B) Biochemical analysis of D‐lactic acid in the blood of rats. (C) The protein expression of ZO‐1 and PV‐1 in rat intestinal tissues was detected by Western blot analysis. (D) The permeation of dextran in the CSF of rats was examined using a 70‐kDa FITC‐dextran permeation assay. (E) Determination of IL‐6, TNF‐α, and IL‐1β in CSF of rats by ELISA. (F) Expression of PV‐1, ZO‐1, and CD31 in the choroid plexus of rats was detected by immunofluorescence. (*n* = 6 per group). Data are shown as the mean ± SEM. The results were analyzed by an unpaired *t*‐test. ***p < 0.001, ****p < 0.0001.
**Figure S5:** Gavage of AS1842856 alleviates intestinal barrier disruption‐induced BCSFB damage. (A) The intestinal permeability of rats was analyzed by FITC‐BSA permeation assay. (B) Biochemical analysis of D‐lactic acid in the blood of rats. (C) The protein expression of ZO‐1 and PV‐1 in rat intestinal tissues was detected by Western blot analysis. (D) The permeation of dextran in the CSF of rats was examined using a 70‐kDa FITC‐dextran permeation assay. (E) Determination of IL‐6, TNF‐α, and IL‐1β in CSF of rats by ELISA. (F) Expression of PV‐1, ZO‐1, and CD31 in the choroid plexus of rats was detected by immunofluorescence. (*n* = 6 per group). Data are shown as the mean ± SEM. The results were analyzed by an unpaired *t*‐test. ***p < 0.001, ****p < 0.0001.
**Figure S6:** Inhibition of FOXO1 activity reverses the exacerbation of permeability of RIMECs and RChPECs by overexpression of DUSP6. (A) The viability of RIMECs transfected with OE‐DUSP6 and treated with AS1842856, LPS, and THCQ was analyzed using CCK‐8 assays. (B) The permeability of RIMECs was analyzed by FITC‐dextran permeation assay. (C) The protein expression of Occludin, ZO‐1, and PV‐1 in RIMECs was detected by Western blot analysis. (D) Determination of IL‐6, TNF‐α, and IL‐1β in RIMECs by ELISA. (E) Expression of CD31 in RIMECs was detected by immunofluorescence. (F) The viability of RChPECs after culture with CM of RIMECs was analyzed using CCK‐8 assays. (G) The permeability of RChPECs after culture with CM of RIMECs was analyzed by FITC‐dextran permeation assay. (H) The protein expression of Occludin, ZO‐1, and PV‐1 in RChPECs after culture with CM of RIMECs was detected by Western blot analysis. (I) Expression of CD31 in RChPECs after culture with CM of RIMECs was detected by immunofluorescence. (*n* = 5 per group). Data are shown as the mean ± SEM. The results were analyzed by one‐way ANOVA. *p < 0.05, **p < 0.01, ***p < 0.001, ****p < 0.0001.
**Figure S7:** KO‐DUSP6 enhances the protective effect of THCQ on the permeability of RIMECs and attenuates RChPEC loss. (A) DUSP6 protein expression after knockout of DUSP6 in RIMECs was detected by Western blot analysis. (B) The viability of RIMECs infected with KO‐NC or KO‐DUSP6 was analyzed using CCK‐8 assays. (C) The permeability of RIMECs was analyzed by FITC‐dextran permeation assay. (D) The protein expression of Occludin, ZO‐1, and PV‐1 in RIMECs was detected by Western blot analysis. (E) Determination of IL‐6, TNF‐α, and IL‐1β in RIMECs by ELISA. (F) Expression of CD31 in RIMECs was detected by immunofluorescence. (G) The viability of RChPECs after culture with CM of RIMECs was analyzed using CCK‐8 assays. (H) The permeability of RChPECs after culture with CM of RIMECs was analyzed by FITC‐dextran permeation assay. (I) The protein expression of Occludin, ZO‐1, and PV‐1 in RChPECs after culture with CM of RIMECs was detected by Western blot analysis. (J) Expression of CD31 in RChPECs after culture with CM of RIMECs was detected by immunofluorescence. (*n* = 5 per group). Data are shown as the mean ± SEM. The results were analyzed by an unpaired *t*‐test. *p < 0.05, **p < 0.01, ***p < 0.001, ****p < 0.0001.

**Appendix S1:** Supplementary methods.

**Table S1:** Identification of the main components of THCQ (positive ion mode).
**Table S2:** Identification of the main components of THCQ (negative ion mode).

## Data Availability

The data that support the findings of this study are available on request from the corresponding author. The data are not publicly available due to privacy or ethical restrictions.

## References

[1] R. Sonneville , S. Benghanem , L. Jeantin , et al., “The Spectrum of Sepsis‐Associated Encephalopathy: A Clinical Perspective,” Critical Care 27, no. 1 (2023): 386. 37798769 10.1186/s13054-023-04655-8 PMC10552444

[2] Q. Gao and M. S. Hernandes , “Sepsis‐Associated Encephalopathy and Blood‐Brain Barrier Dysfunction,” Inflammation 44, no. 6 (2021): 2143–2150. 34291398 10.1007/s10753-021-01501-3 PMC11044530

[3] K. Krzyzaniak , R. Krion , A. Szymczyk , E. Stepniewska , and M. Sieminski , “Exploring Neuroprotective Agents for Sepsis‐Associated Encephalopathy: A Comprehensive Review,” International Journal of Molecular Sciences 24, no. 13 (2023): 10780. 37445958 10.3390/ijms241310780 PMC10342126

[4] Y. Hong , P. Chen , J. Gao , Y. Lin , L. Chen , and X. Shang , “Sepsis‐Associated Encephalopathy: From Pathophysiology to Clinical Management,” International Immunopharmacology 124, no. Pt A (2023): 110800. 37619410 10.1016/j.intimp.2023.110800

[5] S. C. Tauber , M. Djukic , J. Gossner , H. Eiffert , W. Bruck , and R. Nau , “Sepsis‐Associated Encephalopathy and Septic Encephalitis: An Update,” Expert Review of Anti‐Infective Therapy 19, no. 2 (2021): 215–231. 32808580 10.1080/14787210.2020.1812384

[6] X. Li , X. Xu , J. Zhang , et al., “Review of the Therapeutic Effects of Traditional Chinese Medicine in Sepsis‐Associated Encephalopathy,” Journal of Ethnopharmacology 334 (2024): 118588. 39029543 10.1016/j.jep.2024.118588

[7] S. Zhou , Z. Ai , W. Li , et al., “Deciphering the Pharmacological Mechanisms of Taohe‐Chengqi Decoction Extract Against Renal Fibrosis Through Integrating Network Pharmacology and Experimental Validation In Vitro and In Vivo,” Frontiers in Pharmacology 11 (2020): 425. 32372953 10.3389/fphar.2020.00425 PMC7176980

[8] S. M. Lu , B. Yang , Z. B. Tan , et al., “TaoHe ChengQi Decoction Ameliorates Sepsis‐Induced Cardiac Dysfunction Through Anti‐Ferroptosis via the Nrf2 Pathway,” Phytomedicine 129 (2024): 155597. 38643713 10.1016/j.phymed.2024.155597

[9] S. Carloni , A. Bertocchi , S. Mancinelli , et al., “Identification of a Choroid Plexus Vascular Barrier Closing During Intestinal Inflammation,” Science 374, no. 6566 (2021): 439–448. 34672740 10.1126/science.abc6108

[10] W. Muranyi , C. Schwerk , R. Herold , et al., “Immortalized Human Choroid Plexus Endothelial Cells Enable an Advanced Endothelial‐Epithelial Two‐Cell Type In Vitro Model of the Choroid Plexus,” iScience 25, no. 6 (2022): 104383. 35633941 10.1016/j.isci.2022.104383 PMC9133638

[11] H. F. Chen , H. C. Chuang , and T. H. Tan , “Regulation of Dual‐Specificity Phosphatase (DUSP) Ubiquitination and Protein Stability,” International Journal of Molecular Sciences 20, no. 11 (2019): 2668. 31151270 10.3390/ijms20112668 PMC6600639

[12] L. Ye , J. Huang , X. Liang , et al., “Jiawei Taohe Chengqi Decoction Attenuates CCl(4) Induced Hepatic Fibrosis by Inhibiting HSCs Activation via TGF‐beta1/CUGBP1 and IFN‐Gamma/Smad7 Pathway,” Phytomedicine 133 (2024): 155916. 39094440 10.1016/j.phymed.2024.155916

[13] Z. Yuan , H. Qiao , Z. Wang , et al., “Taohe Chengqi Decoction Alleviated Metabolic‐Associated Fatty Liver Disease by Boosting Branched Chain Amino Acids Catabolism in the Skeletal Muscles of Type 2 Diabetes Mellitus,” Phytomedicine 126 (2024): 155315. 38387274 10.1016/j.phymed.2023.155315

[14] Z. Chun‐Peng , C. Tian , and Y. Xue , “Pharmacological Mechanisms of Taohe Chengqi Decoction in Diabetic Cardiovascular Complications: A Systematic Review, Network Pharmacology and Molecular Docking,” Heliyon 10, no. 13 (2024): e33308. 39044965 10.1016/j.heliyon.2024.e33308 PMC11263673

[15] R. P. Yang , D. K. Cai , Y. X. Chen , et al., “Metabolic Insight Into the Neuroprotective Effect of Tao‐He‐Cheng‐Qi (THCQ) Decoction on ICH Rats Using Untargeted Metabolomics,” Frontiers in Pharmacology 12 (2021): 636457. 34012394 10.3389/fphar.2021.636457 PMC8126979

[16] M. Deng , S. Chen , J. Wu , et al., “Exploring the Anti‐Inflammatory and Immune Regulatory Effects of Taohe Chengqi Decoction in Sepsis‐Induced Lung Injury,” Journal of Ethnopharmacology 333 (2024): 118404. 38824977 10.1016/j.jep.2024.118404

[17] X. Peng , Z. Luo , S. He , L. Zhang , and Y. Li , “Blood‐Brain Barrier Disruption by Lipopolysaccharide and Sepsis‐Associated Encephalopathy,” Frontiers in Cellular and Infection Microbiology 11 (2021): 768108. 34804998 10.3389/fcimb.2021.768108 PMC8599158

[18] S. Hasegawa‐Ishii , M. Inaba , and A. Shimada , “Widespread Time‐Dependent Changes in Tissue Cytokine Concentrations in Brain Regions During the Acute Phase of Endotoxemia in Mice,” Neurotoxicology 76 (2020): 67–74. 31628962 10.1016/j.neuro.2019.10.006

[19] V. V. Giridharan , J. S. Generoso , L. Lence , et al., “A Crosstalk Between Gut and Brain in Sepsis‐Induced Cognitive Decline,” Journal of Neuroinflammation 19, no. 1 (2022): 114. 35606817 10.1186/s12974-022-02472-4 PMC9125851

[20] T. Zhang , L. Chen , G. Kueth , et al., “Lactate's Impact on Immune Cells in Sepsis: Unraveling the Complex Interplay,” Frontiers in Immunology 15 (2024): 1483400. 39372401 10.3389/fimmu.2024.1483400 PMC11449721

[21] F. Dabbagh , H. Schroten , and C. Schwerk , “In Vitro Models of the Blood‐Cerebrospinal Fluid Barrier and Their Applications in the Development and Research of (Neuro)Pharmaceuticals,” Pharmaceutics 14, no. 8 (2022): 1729. 36015358 10.3390/pharmaceutics14081729 PMC9412499

[22] O. Cousins , A. Hodges , J. Schubert , et al., “The Blood‐CSF‐Brain Route of Neurological Disease: The Indirect Pathway Into the Brain,” Neuropathology and Applied Neurobiology 48, no. 4 (2022): e12789. 34935179 10.1111/nan.12789

[23] C. Schwerk and H. Schroten , “In Vitro Models of the Choroid Plexus and the Blood‐Cerebrospinal Fluid Barrier: Advances, Applications, and Perspectives,” Human Cell 37, no. 5 (2024): 1235–1242. 39103559 10.1007/s13577-024-01115-5 PMC11341628

[24] C. S. Chang , Y. C. Liao , C. T. Huang , et al., “Identification of a Gut Microbiota Member That Ameliorates DSS‐Induced Colitis in Intestinal Barrier Enhanced Dusp6‐Deficient Mice,” Cell Reports 37, no. 8 (2021): 110016. 34818535 10.1016/j.celrep.2021.110016

[25] M. Sun , Q. Zheng , L. Wang , et al., “Alcohol Consumption During Adolescence Alters the Cognitive Function in Adult Male Mice by Persistently Increasing Levels of DUSP6,” Molecular Neurobiology 61, no. 6 (2024): 3161–3178. 37978157 10.1007/s12035-023-03794-x

[26] Z. Wu , P. Jiao , X. Huang , et al., “MAPK Phosphatase‐3 Promotes Hepatic Gluconeogenesis Through Dephosphorylation of Forkhead Box O1 in Mice,” Journal of Clinical Investigation 120, no. 11 (2010): 3901–3911. 20921625 10.1172/JCI43250 PMC2964981

[27] M. M. Mori Sequeiros Garcia , J. M. Cohen Sabban , M. A. Dattilo , et al., “Angiotensin II‐Upregulated MAP Kinase Phosphatase‐3 Modulates FOXO1 and p21 in Adrenocortical H295R Cells,” Heliyon 6, no. 3 (2020): e03519. 32181392 10.1016/j.heliyon.2020.e03519 PMC7066232

[28] J. Shen , G. Li , Y. Zhu , et al., “Foxo1‐Induced miR‐92b Down‐Regulation Promotes Blood‐Brain Barrier Damage After Ischaemic Stroke by Targeting NOX4,” Journal of Cellular and Molecular Medicine 25, no. 11 (2021): 5269–5282. 33955666 10.1111/jcmm.16537 PMC8178288

[29] L. Zhou , W. Su , Y. Wang , Y. Zhang , Z. Xia , and S. Lei , “FOXO1 Reduces STAT3 Activation and Causes Impaired Mitochondrial Quality Control in Diabetic Cardiomyopathy,” Diabetes, Obesity & Metabolism 26, no. 2 (2024): 732–744. 10.1111/dom.15369 37961034

