# Supplementary material for: Taohe Chengqi Decoction Attenuates Cognitive Impairment in Experimental Sepsis‐Associated Encephalopathy
Source: CNS Neurosci Ther. 2026 Aug 27;32(8):e71106. doi: 10.1002/cns.71106 (PMC13519671; doi:10.1002/cns.71106)
Supplement: Supplementary file 1 [file CNS-32-e71106-s001.docx]

**Table S1:** Identification of the main components of THCQ (Positive ion mode)

| Name | Formula | CAS_num | Annot. DeltaMass [ppm] | Annotation MW | Calc. MW | Relative concentration of substances (μg/ml) | Relative percentage of substances (%) |
| --- | --- | --- | --- | --- | --- | --- | --- |
| 2-Pyrrolidinecarboxylic acid | C5 H9 N O2 | 4305-67-3 | 1.76 | 115.06333 | 115.06353 | 39.46526943 | 15.05% |
| Amygdalin | C20 H27 N O11 | 29883-15-6 | -0.98 | 457.15841 | 457.15796 | 29.6008616 | 11.29% |
| DA9185000 | C12 H10 O S | 945-51-7 | -0.35 | 202.04524 | 202.04516 | 26.12138443 | 9.96% |
| Choline | C5 H13 N O | 62-49-7 | 3.24 | 103.09971 | 103.10005 | 17.95472207 | 6.85% |
| TMN 355 | C21 H14 Cl F N2 O2 | NA | -2.87 | 380.07278 | 380.07169 | 13.25177457 | 5.05% |
| Liquiritigenin | C15 H12 O4 | 578-86-9 | -1.63 | 256.07356 | 256.07314 | 10.89443746 | 4.15% |
| Cinnamaldehyde | C9 H8 O | 104-55-2 | 0.43 | 132.05751 | 132.05757 | 10.7010951 | 4.08% |
| Betaine | C5 H11 N O2 | 107-43-7 | 1.12 | 117.07898 | 117.07911 | 6.891628279 | 2.63% |
| Cianidanol | C15 H14 O6 | 154-23-4 | -1.08 | 290.07904 | 290.07872 | 5.154463963 | 1.97% |
| L-Pyroglutamic acid | C5 H7 N O3 | 98-79-3 | 0.63 | 129.04259 | 129.04267 | 5.067718942 | 1.93% |
| 1-{[(3S,4S,5R)-2,3,4-Trihydroxy-5-(hydroxymethyl)tetrahydro-2-furanyl]methyl}-2-pyrrolidinecarboxylic acid (non-preferred name) | C11 H19 N O7 | 29118-61-4 | -0.97 | 277.11615 | 277.11588 | 5.041949648 | 1.92% |
| N-Methyl-2-AI | C10 H13 N | 24445-44-1 | -0.42 | 147.1048 | 147.10474 | 4.857847216 | 1.85% |
| 18 β-Glycyrrhetintic Acid | C30 H46 O4 | 471-53-4 | -1.07 | 470.33961 | 470.33911 | 4.709353772 | 1.80% |
| Adenosine | C10 H13 N5 O4 | 58-61-7 | -0.62 | 267.09675 | 267.09659 | 4.498578698 | 1.72% |
| Berberine | C20 H17 N O4 | 2086-83-1 | -1.18 | 335.11576 | 335.11536 | 4.467067132 | 1.70% |
| Diammonium glycyrrhizinate | C42 H62 O16 | 79165-06-3 | -0.68 | 822.40379 | 822.40322 | 3.959471589 | 1.51% |
| Apocynin | C9 H10 O3 | 498-02-2 | -0.32 | 166.06299 | 166.06294 | 3.830854147 | 1.46% |
| (+)-Magnoflorine | C20 H23 N O4 | 2141/9/5 | -1.22 | 341.16271 | 341.16229 | 3.738269568 | 1.43% |
| 5-Hydroxy-1-tetralone | C10 H10 O2 | 28315-93-7 | -0.77 | 162.06808 | 162.06795 | 3.70882524 | 1.41% |
| α-Lactose | C12 H22 O11 | 101312-82-7 | -0.94 | 342.11621 | 342.11589 | 3.637008013 | 1.39% |
| trans-3-Indoleacrylic acid | C11 H9 N O2 | 1204-06-4 | -0.28 | 187.06333 | 187.06328 | 3.581200622 | 1.37% |
| Baicalin | C21 H18 O11 | 21967-41-9 | -0.87 | 446.08491 | 446.08452 | 3.520356775 | 1.34% |
| Coumarin | C9 H6 O2 | 91-64-5 | -0.22 | 146.03678 | 146.03675 | 3.344739376 | 1.28% |
| L-Leucine | C6 H13 N O2 | 61-90-5 | 0.66 | 131.09463 | 131.09471 | 3.065866802 | 1.17% |
| L-Phenylalanine | C9 H11 N O2 | 150-30-1 | -0.01 | 165.07898 | 165.07898 | 3.049904122 | 1.16% |
| (+)-Catechin hydrate | C15 H14 O6 | 225937-10-0 | -1.07 | 290.07904 | 290.07873 | 2.828845499 | 1.08% |
| Pseudoephedrine | C10 H15 N O | 90-82-4 | -0.31 | 165.11536 | 165.11531 | 2.827196116 | 1.08% |
| Bis(2,2-dihydroxyethyl) hydrogen phosphate | C4 H11 O8 P | NA | -0.02 | 218.01915 | 218.01915 | 2.740985667 | 1.05% |
| Stachydrine | C7 H13 N O2 | 471-87-4 | -0.24 | 143.09463 | 143.09459 | 2.610125033 | 1.00% |
| L(-)-Pipecolinic acid | C6 H11 N O2 | 3105-95-1 | 0.56 | 129.07898 | 129.07905 | 2.471341517 | 0.94% |
| Oleamide | C18 H35 N O | 301-02-0 | -0.95 | 281.27186 | 281.2716 | 2.407753269 | 0.92% |
| Ononin | C22 H22 O9 | 486-62-4 | -0.86 | 430.12638 | 430.12601 | 1.970259095 | 0.75% |
| Sinapine | C16 H23 N O5 | 18696-26-9 | -1.6 | 309.15762 | 309.15713 | 1.816346766 | 0.69% |
| p-Coumaric acid | C9 H8 O3 | 501-98-4 | -0.01 | 164.04734 | 164.04734 | 1.804506672 | 0.69% |
| (2R)-5-Methoxy-2-methyl-2,3,8,9-tetrahydro-4H-furo[2,3-H]chromen-4-one | C13 H14 O4 | NA | -0.84 | 234.08921 | 234.08901 | 1.71399005 | 0.65% |
| Emodin-3-methyl ether/Physcion | C16 H12 O5 | 521-61-9 | -1.09 | 284.06847 | 284.06816 | 1.708185334 | 0.65% |
| Puerarin | C21 H20 O9 | 3681-99-0 | -0.92 | 416.11073 | 416.11035 | 1.508196488 | 0.58% |
| Dibutyl phthalate | C16 H22 O4 | 84-74-2 | -1.12 | 278.15181 | 278.1515 | 1.374020084 | 0.52% |
| Palmatine | C21 H21 N O4 | 3486-67-7 | -1.02 | 351.14706 | 351.1467 | 1.203259807 | 0.46% |
| Wogonoside | C22 H20 O11 | 51059-44-0 | -0.8 | 460.10056 | 460.10019 | 1.147500592 | 0.44% |
| Decursinol | C14 H14 O4 | 23458-02-8 | -0.81 | 246.08921 | 246.08901 | 1.097586907 | 0.42% |
| D-(+)-Pipecolinic acid | C6 H11 N O2 | 1723-00-8 | 0.86 | 129.07898 | 129.07909 | 1.093860912 | 0.42% |
| Scutellarin | C21 H18 O12 | 27740-01-8 | -0.77 | 462.07983 | 462.07947 | 1.086357562 | 0.41% |
| Isoliquiritin | C21 H22 O9 | 5041-81-6 | -0.86 | 418.12638 | 418.12602 | 1.057761188 | 0.40% |
| 5-Hydroxymethylfurfural | C6 H6 O3 | 67-47-0 | 1.03 | 126.03169 | 126.03182 | 1.046194627 | 0.40% |
| Isoliquiritigenin | C15 H12 O4 | 961-29-5 | -1.16 | 256.07356 | 256.07326 | 1.028037256 | 0.39% |
| 2,5-DMMA | C12 H19 N O2 | 54687-43-3 | -0.6 | 209.14158 | 209.14145 | 0.993712064 | 0.38% |
| Triethyl phosphate | C6 H15 O4 P | 78-40-0 | 0.01 | 182.0708 | 182.0708 | 0.893844787 | 0.34% |
| Resveratrol | C14 H12 O3 | 501-36-0 | -0.98 | 228.07864 | 228.07842 | 0.877194095 | 0.33% |
| 4-Guanidinobutyric acid | C5 H11 N3 O2 | 463-00-3 | -0.03 | 145.08513 | 145.08512 | 0.860964902 | 0.33% |

**Table S2:** Identification of the main components of THCQ (Negative ion mode)

| Name | Formula | CAS_num | Annot. DeltaMass [ppm] | Annotation MW | Calc. MW | Relative concentration of substances (μg/ml) | Relative percentage of substances (%) |
| --- | --- | --- | --- | --- | --- | --- | --- |
| Cefsumide | C17 H20 N4 O6 S2 | 54818-11-0 | 1.11 | 440.08243 | 440.08292 | 505.4929764 | 21.05% |
| Amygdalin | C20 H27 N O11 | 29883-15-6 | -1.97 | 457.15841 | 457.15751 | 419.3848962 | 17.47% |
| Rheic acid | C15 H8 O6 | 478-43-3 | -2.46 | 284.03209 | 284.03139 | 171.196478 | 7.13% |
| Glycyrrhizic acid | C42 H62 O16 | 1405-86-3 | -0.93 | 822.40379 | 822.40302 | 139.8502891 | 5.82% |
| Liquiritin | C21 H22 O9 | 551-15-5 | -1.96 | 418.12638 | 418.12556 | 111.7656092 | 4.65% |
| Isovitexin | C21 H20 O10 | 38953-85-4 | -2.56 | 432.10565 | 432.10454 | 104.8274685 | 4.37% |
| (+)-Catechin hydrate | C15 H14 O6 | 225937-10-0 | -2.2 | 290.07904 | 290.0784 | 93.78196728 | 3.91% |
| Prulaurasin | C14 H17 N O6 | 138-53-4 | -2.97 | 295.10559 | 295.10471 | 78.53819179 | 3.27% |
| Irilone | C16 H10 O6 | 41653-81-0 | -2.54 | 298.04774 | 298.04698 | 73.1925257 | 3.05% |
| N-{[4-(Hydroxyamino)phenyl]sulfonyl}-N-[2-(2-naphthyloxy)ethyl]-beta-D-glucopyranosylamine | C24 H28 N2 O9 S | NA | 1.92 | 520.15155 | 520.15255 | 63.60912066 | 2.65% |
| Liguiritigenin-7-O-β-D-apiosyl-4'-O-β-D-glucoside | C26 H30 O13 | 199796-12-8 | -1.87 | 550.16864 | 550.16761 | 51.33669035 | 2.14% |
| Sucrose | C12 H22 O11 | 57-50-1 | -2.68 | 342.11621 | 342.11529 | 41.72622381 | 1.74% |
| 6-O-[(2E)-3-Phenyl-2-propenoyl]-1-O-(3,4,5-trihydroxybenzoyl)-β-D-glucopyranose | C22 H22 O11 | NA | -1.63 | 462.11621 | 462.11546 | 40.94955563 | 1.71% |
| Apigenin 7-O-glucuronide | C21 H18 O11 | 29741-09-1 | -2.25 | 446.08491 | 446.08391 | 36.05039513 | 1.50% |
| Chrysophanol 8-O-β-D-glucoside | C21 H20 O9 | 13241-28-6 | -1.58 | 416.11073 | 416.11008 | 31.88919466 | 1.33% |
| Ethyl 6-methyl-4-[3-(4-methylphenyl)-1-phenyl-1H-pyrazol-4-yl]-2-thioxo-1,2,3,4-tetrahydro-5-pyrimidinecarboxylate | C24 H24 N4 O2 S | NA | 0.06 | 432.162 | 432.16202 | 29.30194587 | 1.22% |
| (2E,6E)-8-Anilino-3,7-dimethyl-2,6-octadien-1-yl trihydrogen diphosphate | C16 H25 N O7 P2 | NA | 0.45 | 405.11062 | 405.11081 | 29.27232014 | 1.22% |
| Alizarin | C14 H8 O4 | 72-48-0 | -4.14 | 240.04226 | 240.04126 | 28.83790745 | 1.20% |
| Rosmarinic acid | C18 H16 O8 | 20283-92-5 | -1.64 | 360.08452 | 360.08393 | 24.57097879 | 1.02% |
| (15Z)-9,12,13-Trihydroxy-15-octadecenoic acid | C18 H34 O5 | NA | -2.53 | 330.24062 | 330.23979 | 19.71484606 | 0.82% |
| Diosmetin-7-O-β-D-glucopyranoside | C22 H22 O11 | 20126-59-4 | -1.88 | 462.11621 | 462.11534 | 15.7247995 | 0.66% |
| Paeoniflorin | C23 H28 O11 | 23180-57-6 | -2.38 | 480.16316 | 480.16202 | 14.78610755 | 0.62% |
| Liquiritigenin | C15 H12 O4 | 578-86-9 | -2.86 | 256.07356 | 256.07283 | 13.84072683 | 0.58% |
| Isoliquiritin | C21 H22 O9 | 5041-81-6 | -2.18 | 418.12638 | 418.12547 | 13.1430561 | 0.55% |
| Tinnevellin glucoside | C20 H24 O9 | 80358-06-1 | -2.49 | 408.14203 | 408.14102 | 12.22946216 | 0.51% |
| Wogonoside | C22 H20 O11 | 51059-44-0 | -2.01 | 460.10056 | 460.09964 | 11.05485899 | 0.46% |
| Puerarin | C21 H20 O9 | 3681-99-0 | -2.19 | 416.11073 | 416.10982 | 10.97976768 | 0.46% |
| (-)-Epicatechin gallate (-)- | C22 H18 O10 | 1257-08-5 | -2.13 | 442.09 | 442.08905 | 10.63301334 | 0.44% |
| Scutellarin | C21 H18 O12 | 27740-01-8 | -1.68 | 462.07983 | 462.07905 | 10.02039399 | 0.42% |
| Polydatin | C20 H22 O8 | 27208-80-6 | -2.43 | 390.13147 | 390.13052 | 9.669467707 | 0.40% |
| Oroxylin A | C16 H12 O5 | 480-11-5 | -2.82 | 284.06847 | 284.06767 | 9.056622206 | 0.38% |
| Isochlorogenic acid B | C25 H24 O12 | 14534-61-3 | -2.45 | 516.12678 | 516.12551 | 7.814259508 | 0.33% |
| Corchorifatty acid F | C18 H32 O5 | 95341-44-9 | -2.48 | 328.22497 | 328.22416 | 7.632607433 | 0.32% |
| Astragalin | C21 H20 O11 | 480-10-4 | -2.46 | 448.10056 | 448.09946 | 6.694174795 | 0.28% |
| Luteolin 7-glucuronide | C21 H18 O12 | 29741-10-4 | -1.81 | 462.07983 | 462.07899 | 6.241661684 | 0.26% |
| Neohesperidin | C28 H34 O15 | 13241-33-3 | -1.85 | 610.18977 | 610.18864 | 5.700927571 | 0.24% |
| Glycitin | C22 H22 O10 | 40246-10-4 | -2.45 | 446.1213 | 446.1202 | 5.520187269 | 0.23% |
| Sennoside B | C42 H38 O20 | 128-57-4 | -1.31 | 862.19564 | 862.19451 | 5.476283788 | 0.23% |
| luteolin-7-O-[beta-D-glucuronosyl-(1->2)-beta-D-glucuronide] | C27 H26 O18 | NA | -1.51 | 638.11191 | 638.11095 | 5.235562752 | 0.22% |
| Rubiadin | C15 H10 O4 | 117-02-2 | -2.72 | 254.05791 | 254.05722 | 4.585358329 | 0.19% |
| 3,5-Dicaffeoylquinic acid | C25 H24 O12 | 2450-53-5 | -2.01 | 516.12678 | 516.12574 | 4.306165342 | 0.18% |
| Wedelolactone | C16 H10 O7 | 524-12-9 | -2 | 314.04265 | 314.04202 | 4.055096639 | 0.17% |
| Cryptochlorogenic acid | C16 H18 O9 | 905-99-7 | -2.38 | 354.09508 | 354.09424 | 3.944981698 | 0.16% |
| Procyanidin B2 | C30 H26 O12 | 29106-49-8 | -1.68 | 578.14243 | 578.14146 | 3.810931665 | 0.16% |
| Eriodictyol | C15 H12 O6 | 552-58-9 | -2.57 | 288.06339 | 288.06265 | 3.783247765 | 0.16% |
| Naringin | C27 H32 O14 | 10236-47-2 | -2.01 | 580.17921 | 580.17804 | 3.600290841 | 0.15% |
| Hesperidin | C28 H34 O15 | 11013-97-1 | -1.72 | 610.18977 | 610.18872 | 3.500775847 | 0.15% |
| Naringenin | C15 H12 O5 | 480-41-1 | -2.43 | 272.06847 | 272.06781 | 3.308137274 | 0.14% |
| Geniposidic acid | C16 H22 O10 | 27741-01-1 | -2.54 | 374.1213 | 374.12035 | 3.0615188 | 0.13% |
| Baicalin | C21 H18 O11 | 21967-41-9 | -1.98 | 446.08491 | 446.08403 | 3.049121863 | 0.13% |
