# Supplementary material for: Taohe Chengqi Decoction Attenuates Cognitive Impairment in Experimental Sepsis‐Associated Encephalopathy
Source: CNS Neurosci Ther. 2026 Aug 27;32(8):e71106. doi: 10.1002/cns.71106 (PMC13519671; doi:10.1002/cns.71106)
Supplement: Supplementary file 2 [file CNS-32-e71106-s002.docx]

**Supplementary Figure 1**

**Supplementary Figure 1** THCQ alleviates cognitive dysfunction in rats with SAE. (A) Ingredients of THCQ. (B) Positive and negative TIC plots of THCQ identified by HPLC-MS/MS. (C) Timeline of the THCQ treatment and CLP modeling in SD rats. (D) The 96-h survival of rats pretreated with THCQ and undergoing sham or CLP surgery was analyzed using the log-rank test. (E) The escape latency of rats in the Morris water maze test. (F) Representative movement trajectories of rats and time spent in the platform quadrant in the exploration test in the Morris water maze test. (G) The residence time of rats in the center of the open field test. (n = 6 per group). Data are shown as the mean ± SEM. The results were analyzed by one-way ANOVA. **p* < 0.05, ***p* < 0.01, ****p* < 0.001, *****p* < 0.0001.

**Supplementary Figure 2**

**Supplementary Figure 2** THCQ attenuates neuronal apoptosis, reduces inflammatory response, and mitigates gut-brain axis-induced CP vascular barrier disruption in septic rats. (A) Expression of Bax and Caspase-3 in the cortex and hippocampus of rats was detected by immunohistochemistry. (B) Expression of iBA1 and GFAP in cortical and hippocampal tissues of rats was detected by immunohistochemistry. (C) The levels of pro-inflammatory cytokines IL-1β, IL-6, and TNF-α in the brain tissues of rats were assessed using ELISA. (D) The levels of ICAM-1 and VCAM-1 in the brain tissues of rats were assessed using ELISA. (E) Expression of PV-1, Claudin-5, and ZO-1 in the choroid plexus of rats was detected by immunofluorescence. (F) Expression of VEGFA in the choroid plexus of rats was detected by immunofluorescence. (G) Expression of CD31 in the choroid plexus of rats was detected by immunofluorescence. (n = 6 per group). Data are shown as the mean ± SEM. The results were analyzed by one-way ANOVA. ***p* < 0.01, ****p* < 0.001, *****p* < 0.0001.

**Supplementary Figure 3**

**Supplementary Figure 3** Protective effect of THCQ on the intestinal barrier is associated with inhibition of DUSP6. (A) The volcano maps of differentially expressed genes screened out from RNS-seq analysis using intestinal tissues of CLP-induced and THCQ-treated rats (n = 3). (B) The intersection of targets of THCQ ingredients predicted by the HERB database and the differentially expressed genes in intestinal tissues. (C) The heatmap of 42 intersecting genes. (D) Expression and location of DUSP6 in microvascular endothelial cells of rat intestinal tissues were detected by dual-labeling immunofluorescence (n = 6). (E) The mRNA expression of DUSP6 in the intestinal tissues of rats was examined using RT-qPCR (n = 6). (F) The protein expression of DUSP6 in RIMECs was detected by Western blot analysis (n = 5). (G) The mRNA expression of DUSP6 in RIMECs was detected by RT-qPCR (n = 5). Data are shown as the mean ± SEM. The results were analyzed by one-way ANOVA. ****p* < 0.001, *****p* < 0.0001.

**Supplementary Figure 4**

**Supplementary Figure 4** Specific overexpression of DUSP6 in endothelial cells impairs the protective effect of THCQ against intestinal barrier disruption. (A) The intestinal permeability of rats was analyzed by FITC-BSA permeation assay. (B) Biochemical analysis of D-lactic acid in the blood of rats. (C) The protein expression of ZO-1 and PV-1 in rat intestinal tissues was detected by Western blot analysis. (D) The permeation of dextran in the CSF of rats was examined using a 70-kDa FITC-dextran permeation assay. (E) Determination of IL-6, TNF-α, and IL-1β in CSF of rats by ELISA. (F) Expression of PV-1, ZO-1, and CD31 in the choroid plexus of rats was detected by immunofluorescence. (n = 6 per group). Data are shown as the mean ± SEM. The results were analyzed by an unpaired t-test. ****p* < 0.001, *****p* < 0.0001.

**Supplementary Figure 5**

**Supplementary Figure 5** Gavage of AS1842856 alleviates intestinal barrier disruption-induced BCSFB damage. (A) The intestinal permeability of rats was analyzed by FITC-BSA permeation assay. (B) Biochemical analysis of D-lactic acid in the blood of rats. (C) The protein expression of ZO-1 and PV-1 in rat intestinal tissues was detected by Western blot analysis. (D) The permeation of dextran in the CSF of rats was examined using a 70-kDa FITC-dextran permeation assay. (E) Determination of IL-6, TNF-α, and IL-1β in CSF of rats by ELISA. (F) Expression of PV-1, ZO-1, and CD31 in the choroid plexus of rats was detected by immunofluorescence. (n = 6 per group). Data are shown as the mean ± SEM. The results were analyzed by an unpaired t-test. ****p* < 0.001, *****p* < 0.0001.

**Supplementary Figure 6**

**Supplementary Figure 6** Inhibition of FOXO1 activity reverses the exacerbation of permeability of RIMECs and RChPECs by overexpression of DUSP6. (A) The viability of RIMECs transfected with OE-DUSP6 and treated with AS1842856, LPS, and THCQ was analyzed using CCK-8 assays. (B) The permeability of RIMECs was analyzed by FITC-dextran permeation assay. (C) The protein expression of Occludin, ZO-1, and PV-1 in RIMECs was detected by Western blot analysis. (D) Determination of IL-6, TNF-α, and IL-1β in RIMECs by ELISA. (E) Expression of CD31 in RIMECs was detected by immunofluorescence. (F) The viability of RChPECs after culture with CM of RIMECs was analyzed using CCK-8 assays. (G) The permeability of RChPECs after culture with CM of RIMECs was analyzed by FITC-dextran permeation assay. (H) The protein expression of Occludin, ZO-1, and PV-1 in RChPECs after culture with CM of RIMECs was detected by Western blot analysis. (I) Expression of CD31 in RChPECs after culture with CM of RIMECs was detected by immunofluorescence. (n = 5 per group). Data are shown as the mean ± SEM. The results were analyzed by one-way ANOVA. **p* < 0.05, ***p* < 0.01, ****p* < 0.001, *****p* < 0.0001.

**Supplementary Figure 7**

**Supplementary Figure 7** KO-DUSP6 enhances the protective effect of THCQ on the permeability of RIMECs and attenuates RChPEC loss. (A) DUSP6 protein expression after knockout of DUSP6 in RIMECs was detected by Western blot analysis. (B) The viability of RIMECs infected with KO-NC or KO-DUSP6 was analyzed using CCK-8 assays. (C) The permeability of RIMECs was analyzed by FITC-dextran permeation assay. (D) The protein expression of Occludin, ZO-1, and PV-1 in RIMECs was detected by Western blot analysis. (E) Determination of IL-6, TNF-α, and IL-1β in RIMECs by ELISA. (F) Expression of CD31 in RIMECs was detected by immunofluorescence. (G) The viability of RChPECs after culture with CM of RIMECs was analyzed using CCK-8 assays. (H) The permeability of RChPECs after culture with CM of RIMECs was analyzed by FITC-dextran permeation assay. (I) The protein expression of Occludin, ZO-1, and PV-1 in RChPECs after culture with CM of RIMECs was detected by Western blot analysis. (J) Expression of CD31 in RChPECs after culture with CM of RIMECs was detected by immunofluorescence. (n = 5 per group). Data are shown as the mean ± SEM. The results were analyzed by an unpaired t-test. **p* < 0.05, ***p* < 0.01, ****p* < 0.001, *****p* < 0.0001.
