# Supplementary material for: Taohe Chengqi Decoction Attenuates Cognitive Impairment in Experimental Sepsis‐Associated Encephalopathy
Source: CNS Neurosci Ther. 2026 Aug 27;32(8):e71106. doi: 10.1002/cns.71106 (PMC13519671; doi:10.1002/cns.71106)
Supplement: Supplementary file 3 [file CNS-32-e71106-s003.docx]

**Supplementary methods**

**Preparation of THCQ**

*Semen Persicae* (Peach Seed, 12 g), *Radix et Rhizoma Rhei* (root and rhziome of Sorrel Rhubarb, 12 g), *Ramulus Cinmomi* (Cassia Twig, 6 g), *Radix Glycyrrhizae Preparata* (Prepared Liauorice Root, 6 g), and *Natrii Sulfas1* (Sodium Sulfate, 6 g) were obtained from the Department of Pharmacy, Xiyuan Hospital of China Academy of Chinese Medical Sciences, soaked in distilled water (10 times the volume of all herbs) for 1 h, and heated to boiling for 30 min. After cooling, the mixture was filtered, and the filtrate was evaporated to 100 mL at 60°C under reduced pressure. The concentrated extract was freeze-dried to obtain a lyophilized powder and dissolved in distilled water before use [1].

**HPLC–MS/MS**

The THCQ lyophilized powder was dissolved and centrifuged at 4000 *g* for 10 min at 4°C. The supernatant was diluted 100-fold and supplemented with 10 µL of the internal standard. The assay was performed on a 0.22 µm PTFE filter. The analysis was carried out using the LC-MS (Thermo, Vanquish, Q Exactive HF) instrument platform. Separation was achieved using a Zorbax Eclipse C18 column (1.8 μm × 2.1 mm × 100 mm). The chromatographic separation conditions were as follows: the column temperature was 30℃; the flow rate was 0.3 mL/min. The mobile phase comprised 0.1% formic acid aqueous solution (A) and pure acetonitrile (B). The injection volume was 2 μL, and the temperature of the autosampler was 4℃ [2].

**CLP surgery**

The study protocol was approved by the Medical Ethics Committee of Xiyuan Hospital of China Academy of Chinese Medical Sciences (approval number: 2025XLC095; approval date: 23^th^ June, 2025). SD rats (6-7 weeks old, half females and half males, mean weight 215 g) were acquired from Vital River (Beijing, China). The rats were acclimatized to 22°C, 45-55% humidity, and a 12 h light/dark cycle. Standard rodent food (casein: 14.0000%, corn starch: 46.592%, maltodextrin: 15.5000%, sucrose: 10.0000%, cellulose: 5.0000%, soybean oil: 4.0000%, AIN-93M mineral mix: 3.5000%, AIN-93M vitamin mix: 1.0000%, cholie bitartrate: 0.2500%, cystine: 0.1800%, butylated hydroxytoluene: 0.0008%) (SiPeiFu, Beijing, China) and water were freely accessible. All rats were acclimatized for a period of 10 days before starting the experiments.

The rats were initially anesthetized with 3% isoflurane, and a 3-centimeter midline dissection was then performed to expose the cecum and adjacent intestine. The cecum was meticulously ligated 50% below the ileocecal valve using 3.0 silk sutures. Subsequently, the cecum was perforated once with an 18-gauge needle to induce moderate sepsis. After squeezing the cecum, a small amount of feces was extruded from the puncture site. The cecum was placed back into the abdominal cavity, and the incision was closed with 3.0 silk sutures. In the sham-operated group, rats underwent all the surgeries, but without ligation and puncture of the cecum [3]. Postoperatively, rats were given an intraperitoneal injection of 0.9% NaCl at 20 mL/kg [4] and rewarmed in a cage for 1 h [5]. In the survival rate experiment, the rats were observed every 1 h after the operation, and the total observation period was 96 h. For behavioral tests, six rats were placed in each group to perform the experiments at 10 days postoperatively. For other experiments, the rats were euthanized at 24 h postoperatively.

**Animal treatments**

For THCQ treatment, the rats were gavaged with 2.82 (low), 5.64 (moderate), and 11.27 (high) g/kg of aqueous decoction in a volume of 3 mL once a day for 3 days before CLP surgery [6]. The positive control drug DXM (30 μg/kg, HY-12719, MedChemExpress, Monmouth Junction, NJ, USA, solubility 125 mg/mL in DMSO) was administered by a single intraperitoneal injection of 200 μL 30 min before CLP [7].

For endothelial cell-specific intervention in septic rats, we integrated the overexpression of the DUSP6 plasmid into the adeno-associated virus (AAV)-TIE1 vectors [8]. Twenty-eight days before CLP surgery, AAV vectors (1 × 10^12^/vg) were administered intravenously into rats. For the FOXO1 activity inhibition, we administered AS1842856 (HY-100596, MedChemExpress) at 50 mg/kg (the volume of gavage was 3 mL and the solubility in DMSO was 5 mg/mL) or equal doses of DMSO by gavage 30 min before CLP surgery [9].

**Morris water maze**

Morris Water Maze Experimental Analysis System (XR-XM101, Softmaze, Shanghai, China) was used to analyze spatial and memory abilities in rats. The water maze facility consists of a circular pool (diameter: 1.7 m and height: 0.65 m). The height of the water in the pool was meticulously regulated to a precise level of 0.35 meters, and the pool was methodically partitioned into four quadrants. A platform slightly below the water surface was placed in the third quadrant of the pool (the diameter of the platform was 0.125 m), and the temperature of the water in the pool was kept at about 20°C. In the first 4 days of training, the rats (10 days after CLP) were placed in a pool of water facing the wall and observed to reach the platform. The test ended when the rats reached the platform or when the 120-s time expired. If the rats failed to reach the designated platform within the stipulated time frame (120 s), they were gently guided towards the platform and permitted to remain there for a duration of 10 s. Four trials were conducted daily (20 min between trials), each with four different quadrants as the starting point. At the end of the training, the rats were dried and returned to their cages. After 24 h, the last escape latency determination and exploration experiment were conducted with all rats starting at the same quadrant. The time it took the rats to find the platform was recorded as the escape latency to assess differences in learning ability, and the residence time of the rats in the quadrant where the platform was located was observed after the platform was removed [10]. The swimming track of the rats was recorded using an animal movement trajectory tracking system (Noldus, Beijing, China) to assess the differences in spatial memory ability of the rats.

**Open-field test**

The rats were placed in the central area of the box (1000*1000*400 mm, XR-XZ301, Softmaze), and the entire area was uniformly divided into equal squares. The animal movement trajectory tracking system was employed to track and record the time each rat spent in the central area for five minutes [11]. After the completion of each test, the apparatus was meticulously sanitized with 75% ethanol.

**Measurement of the permeability of mesenteric microvessels**

For rat intestinal permeability analysis, the rats were anesthetized. The mesenteric ileum was exposed and placed on a transparent slide moistened with 37°C saline. Subsequently, FITC-BSA (Q-0036666, Xi'an Qiyue Biology, Xi'an, Shaanxi, China) dissolved in PBS was injected intravenously at 10 mg/kg. After 10 min, fluorescence permeation images were observed using live fluorescence imaging, and the percentage of fluorescence leakage was quantified [12].

**Evans blue dye**

The BBB permeability was assessed with Evans blue dye. Briefly, rats were anesthetized and injected with 2% Evans blue solution (HY-B1102, MedChemExpress) by tail vein, followed by removal of the circulating staining solution by cardiac perfusion with saline. Then, the brain tissue was quickly removed. Evans blue penetration in the brain tissue was observed and photographed, and the brain tissues were homogenized and centrifuged at 6000 *g* for 30 min to read the absorbance value at 620 nm.

**Measurement of BCSFB permeability**

Rats were injected intravenously with 70 kDa FITC-dextran (53471, Sigma-Aldrich, St Louis, MO, USA) 1 h before isolation of the CSF [13]. Subsequently, intracardiac perfusion was conducted using saline to remove labeled dextran. Subsequently, we anesthetized the rats, and the head was fixed. The head and neck were shaved and sterilized, and a longitudinal incision (about 2 cm) was made along the longitudinal axis with a scalpel. The dorsal neck muscles were bluntly separated with scissors. To avoid bleeding, the deepest muscle attached to the bone was scraped with the dorsal part of the scalpel to expose the foramen magnum. CSF samples were collected by direct needle insertion through the foramen magnum to assess BCSFB permeability by measuring fluorescence at λex/λem = 488/520 nm.

**Sample collection**

Rats were euthanized 24 h after surgery, and brain, intestinal tissues, and CSF were collected. Blood was taken by cardiac puncture. Brain and intestinal tissues were formalin-fixed overnight and paraffin-embedded or prepared directly as frozen sections. CSF and blood samples were stored in a -20°C refrigerator.

**HE staining**

Paraffin-embedded sections of brain and intestinal tissues were deparaffinized, rehydrated, stained with hematoxylin for 10 min, differentiated with differentiation solution for 30 s, and washed twice with tap water. After eosin staining for 2 min, the sections were dehydrated quickly, cleared with xylene, and sealed. The histopathological damage of the cerebral cortex and hippocampus was observed under the microscope [14].

**Nissl staining**

Paraffin sections of rat brain tissues were deparaffinized using xylene, rehydrated using ethanol, and rinsed in distilled water for 2 min. Following the protocol for the Nissl stain (E607316, Sangon, Shanghai, China), the sections were incubated with the Nissl stain solution at room temperature (RT) for 30 min and washed in distilled water for 2 min. After dehydration using ethanol and transparency through xylene, the slices were sealed, and the number of Nissl bodies in the cerebral cortex and hippocampal tissues was counted under the microscope.

**Analysis of apoptosis by TUNEL assay**

Rat brain sections that were deparaffinized and rehydrated were incubated with proteinase K for 30 min at 37°C, followed by treatment with TdT enzyme reaction solution for 1 h at 37°C in the dark as per the TUNEL Apoptosis Detection Kit (TRITC Red Fluorescent Labeling Assay) (BL644A, Biosharp, Hefei, Anhui, China). The sections were incubated with Streptavidin-TRITC working solution in the dark at 37°C for 30 min. After staining the nuclei of the cells with DAPI, the sections were subsequently sealed to observe the number of apoptotic cells in the cerebral cortex and hippocampal tissues. The number of apoptotic cells was counted by experimentally uninformed observers, and the number of positively stained nuclei in the high-magnification field of view was counted for each sample [15].

**Immunohistochemical assay**

Paraffin-embedded brain sections were deparaffinized, rehydrated, and boiled in pH 6.0 citrate buffer. The sections were treated with 3% hydrogen peroxide solution for 10 min at RT and sealed in 1% BSA solution for 30 min [16]. After that, the sections were probed with primary antibodies to iBA1 (1:50, A19776, ABclonal, Wuhan, Hubei, China), GFAP (1:1000, ab7260, Abcam, Cambridge, UK), myeloperoxidase (MPO, 1:50, MPO-101AP, Thermo Fisher Scientific Inc., Waltham, MA, USA), Bax (1:50, A19684, Abclonal), and Caspase-3 (1:100, A11319, Abclonal) at 4°C overnight and with goat anti-rabbit IgG antibody (HRP) (1:100, GTX213110-01, GeneTex, Inc., Alton Pkwy, Irvine, CA, USA) at RT for 1 h. A DAB solution was applied for 3 min, followed by hematoxylin staining for 30 s. Subsequently, the sections were dehydrated, cleared, and sealed. The cerebral cortex and hippocampal tissues were viewed under a microscope.

**RNA-Seq analysis**

Briefly, TRIzol reagent was used to extract total RNA from rat intestinal tissues (n = 3), and RNA integrity was assessed after examining RNA quality. Transcriptome library preparation was performed using the QIAseq Stranded RNA Lib Kit UDI (24) (180450, Qiagen, Valencia, CA, USA). After enrichment and quantification of PCR products, sequencing was performed on a Novaseq 6000 sequencer [17]. The R software (v.4.2.2) package “DESeq2” (v.1.40.0) was used for differential expression analysis with the screening criterion: adjusted *p-value* < 0.05. The results were then visualized using the R package “ggpubr” (v. 0.4.0).

**Extraction of rat intestinal microvascular endothelial cells (RIMECs)**

Intestinal tissues were obtained from 5-week-old SD rats (Vital River) as previously described [18], cut longitudinally, and rinsed with PBS containing a 5% penicillin-streptomycin mixture. The intestinal tissues were cut into 1-cm pieces, stirred with 0.05% trypsin for 10 min at 37°C to remove epithelial cells, cut into 1-mm pieces, and detached at 37°C for 15 min with the addition of 2% type II collagenase, followed by filtering through a 400-mesh sieve. The filtrate was centrifuged at 400 *g* for 5 min at 4°C. The cells were cultured in high-glucose DMEM (PM150210B, Procell, Wuhan, Hubei, China) supplemented with 20% FBS, 0.05 mg/mL ECGS, and 1% penicillin-streptomycin mixture at 37°C and 5% CO_2_. After the cells had adhered to the wells, the medium was refreshed. Primary RIMECs were finally obtained.

**Extraction of rat choroid plexus endothelial cells (RChPECs)**

Cells were isolated from the choroid plexus using a method similar to that described in a previous report [19]. Fifty newborn rat pups (24-h) were euthanized using 150 mg/kg of sodium pentobarbital. After cutting along the midline of the skull and removing the whole brain, the lateral ventricles, third, and fourth ventricles were localized by microscopy on ice. The choroid plexus was gently peeled using microforceps, and the blood cells were removed after three washes with PBS. Next, the isolated choroid plexus tissue was sheared to 1 mm^3^ and digested with a digestive solution mixed with collagenase II + trypsin for 30 min at 37°C. Digestion was terminated by DMEM/F12 medium containing 20% FBS. The undigested tissue was removed by filtering sequentially through a 70 μm and a 40 μm cell sieve. The supernatant was removed by centrifugation at 300 *g* for 5 min. The cells were seeded into gelatin-coated Petri dishes and cultured at 37°C, 5% CO_2_. The culture medium was refreshed every 2 days. The cells observed to be in typical cobblestone-like arrangements were regarded as RChPECs. We finally identified cell purity by analyzing the positive expression of CD31 in cells using FITC-CD31 (MA5-16952, Thermo Fisher).

**Cell transfection and treatment**

Plasmids overexpressing DUSP6 were acquired from Genechem (Shanghai, China). RIMECs were seeded into 96-well plates, and the overexpression plasmid was transfected into the cells using Lipofectamine 3000 (Thermo Fisher). After 48 h, the medium was refreshed, and the transfection efficiency was substantiated by detecting the expression of DUSP6 using RT-qPCR. For intracellular DUSP6 knockdown, a rat Dusp6 CRISPR lentiviral vector (guide sequence: CGCGCTATTCACGCGCTGCG) was obtained from VectorBuilder (Guangzhou, Guangdong, China). After infecting the cells with the lentiviral vector, we screened the positive cells with puromycin to obtain the stable knockout cells.

To mimic sepsis-induced intestinal barrier disruption *in vitro*, RIMECs were induced with 10 μg/mL lipopolysaccharides (LPS, S7850, Selleck, Houston, TX, USA) for 24 h [20]. For the *in vitro* treatment of THCQ, we dissolved the THCQ lyophilized powder and treated the cells with THCQ at a concentration of 100 μg/mL in parallel with LPS treatment. PBS was used as the control for both LPS and RIMECs. For the inhibition of FOXO1 activity in cells, we pretreated cells with 10 µM AS1842856 for 24 h before LPS exposure. The control group was treated with equal doses of DMSO.

To clarify the effect of the altered permeability of RIMECs on the permeability of RChPECs, we centrifuged the RIMECs at 1000 *g* for 10 min at the end of each treatment to collect conditioned medium (CM) and cultured the RChPECs for 24 h using the CM.

**Cell viability assay**

RIMECs and RChPECs were seeded into 96-well plates for pre-culturing. According to the manufacturer’s instructions for the CCK-8 Cell Proliferation and Cytotoxicity Assay Kit (CCK-8, CA1210, Solarbio, Beijing, China), the cells after various treatments were incubated with 10 µL of CCK-8 solution at 37°C, 5% CO_2_ for 4 h. Absorbance was measured at 450 nm. Cell viability was defined as the percentage (%) of the absorbance of the treatment group relative to that of the control group.

**Cell permeability assessment**

RIMECs and RChPECs were seeded into the apical chamber of Transwell at 1 × 10^4^ cells/well and cultured for 72 h until the cells were confluent. The medium was removed, and the apical chamber cells were incubated with 10 mg/mL FITC-dextran for 1 h [21]. At the end of the incubation, the absorbance values of the basolateral chamber solutions at 492 nm (excitation) and 520 nm (emission) wavelengths were read using the microplate reader to assess changes in cell permeability.

**Subcellular fractionation**

According to the instructions of NE-PER Nuclear and Cytoplasmic Extraction Reagent (78835, Thermo Fisher), rat intestinal tissues were cut into small pieces and centrifuged at 500 *g* for 5 min. The tissues were homogenized in CER I and incubated on ice for 1 min with CER II, followed by centrifugation at 16,000 *g* for 5 min. The supernatant was harvested as a cytoplasmic extract. The remaining nuclear precipitate was suspended in NER and vortexed on ice for 40 min. Centrifugation was performed in a centrifuge at 16,000 *g* for 10 min, and the supernatant was harvested as the cell nucleus extract. Nuclear translocation was assessed by western blot detection of FOXO1 expression in nuclear and cytoplasmic fractions, with GAPDH as the cytoplasmic reference and Histone H3 as the nuclear reference.

**Co-immunoprecipitation assays (****Co-IP)**

Co-IP assays were conducted as previously described [22]. Briefly, RIMECs were lysed on ice for 30 min and centrifuged at 12,000 *g* for 10 min. The supernatants were incubated with anti-DUSP6 (1:50, ab76310, Abcam), anti-FOXO1 (1:100, MA5-14846, Thermo Fisher), or rabbit IgG control antibody (30000-0-AP, ProteinTech Group, Chicago, IL, USA) overnight at 4°C on a rotator, followed by another 2-h incubation with 30 μL of pre-washed protein A/G agarose beads. After sufficient washing with diluted lysis buffer, the immunoprecipitated complexes were resuspended in SDS-PAGE buffer for western blot analysis.

**Dual-luciferase assay**

To assess the transcriptional activity of FOXO1, we obtained pFOXO1-TA-Luc (reporter gene plasmid) (D4085-100 μg, Beyotime) and transfected the reporter plasmid or the FOXO1 ΔSer256 plasmid into RIMECs overexpressing DUSP6 using Lipofectamine 3000. After 48 h, the dual-luciferase reporter assay system (E1910, Promega Corporation, Madison, WI, USA) was used for analysis.

**Western blot analyses**

Total protein was extracted from tissues or RIMECs using RIPA lysis buffer, and the protein concentration of each extract was determined using the BCA protein assay. Next, equal amounts of protein from each extract were separated by 12% SDS-PAGE, and the proteins were transferred to PVDF membranes, which were incubated for 2 h with 5% skim milk. The primary antibodies used included Occludin (1:1000, #91131, CST, Beverly, MA, USA), Claudin-5 (1:1000, 35-2500, Thermo Fisher), ZO-1 (1:1000, 40-2300, Thermo Fisher), VEGFA (1:2000, 19003-1-AP, ProteinTech Group), PV-1 (1:1000, 4452-1-AP, ProteinTech Group), p-FOXO1 (Ser256) (1:500, bs-3142R, Bioss Biotech, Beijing, China), FOXO1 (1:1000, MA5-14846, Thermo Fisher), Β-tubulin (1:500, ab6046, Abcam), Histone H3 (1:1000, ab1791, Abcam), and GAPDH (1:2000, AC027, Abclonal). The PVDF membranes were re-probed with diluted primary antibodies at 4℃ overnight. The HRP-conjugated secondary antibodies IgG (65-6120 or 62-6520, Thermo Fisher) were incubated at RT for 2 h. Protein bands were visualized using an ECL detection system [23] and quantified with ImageJ software.

**ELISA**

Blood, CSF, brain tissue from each group of rats, and supernatant samples of RIMEC CM were collected to detect cytokines using rat IL-6 ELISA kit (E-EL-R0015, Elabscience, Wuhan, Hubei, China), rat IL-1β ELISA kit (E-HSEL-R0002, Elabscience), rat TNF-alpha Quantikine ELISA kit (RTA00, R&D Systems, Minneapolis, MN, USA), rat ICAM-1/CD54 Quantikine ELISA kit (RIC100, R&D Systems), and rat VCAM-1 ELISA kit (E-EL-R1061, Elabscience). All operations were performed in accordance with the manufacturer's instructions. Briefly, the plate was incubated with samples or standards to be tested for 90 min, with biotinylated antibody working solution for 1 h, with HRP enzyme-conjugated working solution for 30 min, and with substrate solution for 15 min in the dark (all at 37℃). The reaction termination solution was added, and the absorbance value was read at 450 nm. The content of the sample to be tested was calculated from the standard curve based on the absorbance value of the standard.

**Biochemical analysis**

D-Lactic Acid (D-LA) content assay kit (BC5350, Solarbio) was used to detect D-LA in rat blood samples. All operations were performed in accordance with the manufacturer's instructions. The rat blood samples were centrifuged with extract I at 12,000 *g* for 10 min at 4°C. The supernatant was centrifuged again with extract II at 12,000 *g* for 10 min, and the supernatant was harvested as the sample to be tested. Next, various reagents were added to configure the reaction system, and after mixing, the reaction was carried out at 37°C for 30 min in the dark. The reaction solution was transferred to the cuvette to measure the absorbance value at 450 nm, and the content of the sample to be tested was calculated from the absorbance of the standard.

**Immunofluorescence assay**

Frozen sections of brain tissue, RIMECs, and RChPECs were fixed in 4% paraformaldehyde and sealed in 5% goat serum for 2 h. Protein expression was visualized using a method similar to that described in a previous report [24]. The samples were probed with rabbit anti-PV-1 (1:100, 14452-1-AP, ProteinTech Group), mouse anti-Claudin-5 (1:100, 35-2500, Thermo Fisher), rabbit anti-ZO-1 (1:1000, 40-2200, Thermo Fisher), rabbit anti-VEGFA (1:200, 19003-1-AP, ProteinTech Group), rabbit anti-FOXO1 (1:50, MA5-14846, Thermo Fisher), mouse anti-FOXO1 (1:200, GTX60558, GeneTex), mouse anti-CD31 (1:100, MA1-80069, Thermo Fisher), and rabbit anti-DUSP6 (1:50, ab238512, Abcam) at 4°C overnight. The secondary antibodies, goat anti-rabbit IgG H&L (1:200, ab150079 or ab150077, Abcam) and goat anti-mouse IgG H&L (1:200, ab150115 or ab150113, Abcam), were added for a 1-h incubation at RT, following DAPI application. The samples were viewed under a fluorescence microscope.

**RNA isolation and qPCR**

Total RNA was extracted from rat intestinal tissues and RIMECs by TRIzol reagent (Thermo Fisher) and reverse transcribed into cDNA by PrimeScript RT reagent Kit (RR037Q, Takara, Dalian, Liaoning, China). The relative gene expression was determined with the Applied Biosystems 7300 using TB Green Premix Ex Taq (Tli RNaseH Plus)(RR420Q, Takara). The 2^−ΔΔCT^ method was used to calculate fold changes [25], with GAPDH as the reference. The RT-qPCR are listed below: DUSP6 Forward: 5′-GGCTGGCCGAGAGAATGTAT-3′, DUSP6 Reverse: 5′-CACCACAGTTTTTGCCTCGG-3′, GAPDH Forward: 5′-GCGAGATCCCGCTAACATCA-3′, DUSP6 Reverse: 5′-ATTCGAGAGAAGGGAGGGCT-3′.

**Statistical analysis**

The experimental data were analyzed statistically using the GraphPad Prism software (10.1.2, GraphPad, San Diego, CA, USA). Results were presented as mean ± SEM. For comparisons between two continuous variables, the unpaired t-test was applied. One-way ANOVA or two-way repeated measures ANOVA tests, followed by Tukey's tests, were used for comparisons among more than two groups. Survival curves were calculated using the Kaplan-Meier method, and survival analysis was performed using the log-rank test. Statistical significance was defined as a *p*-value of < 0.05.

**References**

1. Deng M, Chen S, Wu J, Su L, Xu Z, Jiang C*, et al.* Exploring the anti-inflammatory and immune regulatory effects of Taohe Chengqi decoction in sepsis-induced lung injury. J Ethnopharmacol 2024, 333: 118404.

2. Lim Y, Kim A, Lee YM, Cho H. Development of a sensitive LC-MS/MS method for determination of N-nitrosopiperazine in levocetirizine. Anal Methods 2024, 16: 6494-6500.

3. Giridharan VV, Generoso JS, Lence L, Candiotto G, Streck E, Petronilho F*, et al.* A crosstalk between gut and brain in sepsis-induced cognitive decline. J Neuroinflammation 2022, 19: 114.

4. Tan L, She H, Zheng J, Peng X, Guo N, Zhang B*, et al.* Effects of Malate Ringer's solution on myocardial injury in sepsis and enforcement effects of TPP@PAMAM-MR. J Transl Med 2022, 20: 591.

5. Liang H, Song H, Zhang X, Song G, Wang Y, Ding X*, et al.* Metformin attenuated sepsis-related liver injury by modulating gut microbiota. Emerg Microbes Infect 2022, 11: 815-828.

6. Yang RP, Cai DK, Chen YX, Gang HN, Wei M, Zhu DQ*, et al.* Metabolic Insight Into the Neuroprotective Effect of Tao-He-Cheng-Qi (THCQ) Decoction on ICH Rats Using Untargeted Metabolomics. Front Pharmacol 2021, 12: 636457.

7. Zhang S, Feng X, Yang G, Tan H, Cheng X, Tang Q*, et al.* Dexmedetomidine ameliorates acute kidney injury by regulating mitochondrial dynamics via the alpha2-AR/SIRT1/PGC-1alpha pathway activation in rats. Mol Med 2024, 30: 184.

8. She H, Tan L, Du Y, Zhou Y, Guo N, Zhang J*, et al.* VDAC2 malonylation participates in sepsis-induced myocardial dysfunction via mitochondrial-related ferroptosis. Int J Biol Sci 2023, 19: 3143-3158.

9. Yan D, Cai Y, Luo J, Liu J, Li X, Ying F*, et al.* FOXO1 contributes to diabetic cardiomyopathy via inducing imbalanced oxidative metabolism in type 1 diabetes. J Cell Mol Med 2020, 24: 7850-7861.

10. Ge C, Chen W, Zhang L, Ai Y, Zou Y, Peng Q. Chemogenetic activation of the HPC-mPFC pathway improves cognitive dysfunction in lipopolysaccharide -induced brain injury. Theranostics 2023, 13: 2946-2961.

11. Du X, Yin M, Yuan L, Zhang G, Fan Y, Li Z*, et al.* Reduction of depression-like behavior in rat model induced by ShRNA targeting norepinephrine transporter in locus coeruleus. Transl Psychiatry 2020, 10: 130.

12. Hou D, Liu R, Hao S, Dou Y, Chen G, Liu L*, et al.* Notoginsenoside R1 improves intestinal microvascular functioning in sepsis by targeting Drp1-mediated mitochondrial quality imbalance. Pharm Biol 2024, 62: 250-260.

13. Yarnitsky D, Gross Y, Lorian A, Shalev A, Lamensdorf I, Bornstein R*, et al.* Blood-brain barrier opened by stimulation of the parasympathetic sphenopalatine ganglion: a new method for macromolecule delivery to the brain. J Neurosurg 2004, 101: 303-309.

14. Ates S, Ulger H, Ucar S, Okan A, Ocak M, Guvenilir E*, et al.* Evaluation of the Effects of Thymoquinone on RAGE/NOX4 Expressions and Brain Tissue Morphometry in Experimental Alzheimer's Disease Induced by Amyloid Beta 1-42 Peptide. Biomolecules 2025, 15.

15. Jacob A, Hensley LK, Safratowich BD, Quigg RJ, Alexander JJ. The role of the complement cascade in endotoxin-induced septic encephalopathy. Lab Invest 2007, 87: 1186-1194.

16. Cao Y, Dong YX, Xu J, Chu GL, Yang ZH, Liu YM. Spatiotemporal expression of Nogo-66 receptor after focal cerebral ischemia. Neural Regen Res 2016, 11: 132-136.

17. El-Shehawi AM, El-Shazly S, Ahmed M, Alkafafy M, Sayed S, Farouk S*, et al.* Transcriptome Analysis of Testis from HFD-Induced Obese Rats (Rattus norvigicus) Indicated Predisposition for Male Infertility. Int J Mol Sci 2020, 21.

18. Liu P, Bian Y, Zhong J, Yang Y, Mu X, Liu Z. Establishment and characterization of a rat intestinal microvascular endothelial cell line. Tissue Cell 2021, 72: 101573.

19. Itokazu Y, Kitada M, Dezawa M, Mizoguchi A, Matsumoto N, Shimizu A*, et al.* Choroid plexus ependymal cells host neural progenitor cells in the rat. Glia 2006, 53: 32-42.

20. Zhang HX, Li YY, Liu ZJ, Wang JF. Quercetin effectively improves LPS-induced intestinal inflammation, pyroptosis, and disruption of the barrier function through the TLR4/NF-kappaB/NLRP3 signaling pathway in vivo and in vitro. Food Nutr Res 2022, 66.

21. Jun JH, Park S, Kim JY, Lim JY, Park GT, Kim JH*, et al.* Combination Therapy of Placenta-Derived Mesenchymal Stem Cells with WKYMVm Promotes Hepatic Function in a Rat Model with Hepatic Disease via Vascular Remodeling. Cells 2022, 11.

22. Wang M, Gu Y, Li Q, Feng B, Lv X, Zhang H*, et al.* The Traf2 and NcK interacting kinase inhibitor NCB-0846 suppresses seizure activity involving the decrease of GRIA1. Genes Dis 2024, 11: 100997.

23. Chen S, Tang C, Ding H, Wang Z, Liu X, Chai Y*, et al.* Maf1 Ameliorates Sepsis-Associated Encephalopathy by Suppressing the NF-kB/NLRP3 Inflammasome Signaling Pathway. Front Immunol 2020, 11: 594071.

24. Yao P, Wu L, Yao H, Shen W, Hu P. Acute hyperglycemia exacerbates neuroinflammation and cognitive impairment in sepsis-associated encephalopathy by mediating the ChREBP/HIF-1alpha pathway. Eur J Med Res 2024, 29: 546.

25. Livak KJ, Schmittgen TD. Analysis of relative gene expression data using real-time quantitative PCR and the 2(-Delta Delta C(T)) Method. Methods 2001, 25: 402-408.
